# Measuring the quality of life of students with autism in Chilean general education schools

**DOI:** 10.3389/fpsyt.2026.1790139

**Published:** 2026-05-20

**Authors:** Victoria Sánchez-Gómez, Sebastián Zenteno-Osorio, Antonio M. Amor, Mauricio López-Cruz, Miguel Ángel Verdugo

**Affiliations:** 1 Institute for Community Inclusion (INICO), Univerisity of Salamanca, Salamanca, Spain; 2 Department of Basic Psychology, Pyschobiology, and Behavioral Science Methodology, University of Salamanca, Salamanca, Spain; 3 Facultad de la Salud y Ciencias Sociales, Universidad de Las Américas, Santiago, Chile; 4 Department of Personality, Assessment, and Psychological Treatments, University of Salamanca, Salamanca, Spain; 5 Department of Psychology, University of Chile, Santiago, Chile

**Keywords:** assessment, autism, inclusive education, intellectual and developmental disabilities, quality of life

## Abstract

**Introduction:**

The enrollment of students with autism in the Chilean general education system has increased exponentially, posing challenges for schools, which need useful approaches and strategies to enable them to respond to students and fulfill their aspirations and needs. The Quality of Life Index-Primary Education (QoLI-PE) is an assessment tool, initially developed in Spain, designed to assess the quality of life (QoL) of students with intellectual and developmental disabilities (IDD) in general education. This study aims to evaluate the psychometric properties and potential utility of the QoLI-PE in a population of students with autism enrolled in Chilean general schools. Specific aims included analyzing evidence of validity regarding internal structure, analyzing reliability, and exploring the QoL of students with autism in Chilean general schools.

**Method:**

242 students with autism attending general education schools in four regions of Chile were assessed by key informants using the QoLI-PE. Confirmatory factor analyses (CFA) were conducted, based on the theoretical model and the configuration previously tested in the Spanish context. Both the first-order model and the second-order model were tested. Reliability was reported according to internal consistency. To compare domains at the within-subjects level, results were analyzed using repeated measures ANOVA and *post-hoc* analyses.

**Results:**

The second-order model was selected as the more plausible representation. Overall, the CFA supported the internal structure of the instrument for a model composed of eight domains, in which all items presented high and significant factor loadings. Internal consistency was excellent for all domains. Students with autism scored higher on material well-being and rights, while self-determination, interpersonal relationships, and social inclusion were the areas of greatest concern.

**Discussion:**

The potential utility of the QoLI-PE in Chilean schools is analyzed, specifically in terms of its contribution to the goals of the recent law regulating the inclusion of people with autism. Possible intervention strategies informed by the instrument are discussed as a means to guide and support the inclusion of Chilean students with autism at different levels. Future directions based on similarities in the application of the instrument between Chile and Spain are highlighted.

## 1 Introduction

The right to inclusive education (IE), set forth in Article 24 of the Convention on the Rights of Persons with Disabilities (UNCRPD) (1), establishes that States Parties must provide an inclusive education system that ensures access, participation, learning, and the maximum possible development of students with disabilities to their fullest potential, and on an equal basis with their peers without disabilities. In this understanding of IE, student participation is as important as their learning (2), so States must ensure participation in general education contexts, providing all reasonable adjustments and supports necessary for academic development and also for the social development of students.

Although there have been undeniable advances in IE in recent years—reflected in policies, rights, research trends, and changes in enrollment rates (3–8)— students with intellectual and developmental disabilities (IDD) are still at risk of exclusion: their enrollment rates in the general education system have stagnated, and they continue to represent the majority of enrollment in special education systems (5, 9, 10). In this scenario, it is necessary to adopt approaches that enable general schools to offer more guarantees to students with IDD and promote outcomes related to their access, participation, learning, and maximum possible development in these environments. Two complementary approaches are important in this regard: the supports paradigm (11) and the multidimensional model of individual quality of life (QoL) by Schalock and Verdugo (12); which have converged to form the Quality of Life Supports Model (QoLSM) (13, 14).

The QoLSM brings together the implications of both approaches and offers a conceptual and applied framework that makes it possible—based on positive values regarding people with IDD shared with the UNCRPD and the provisions of its Article 24—to understand the aspirations and needs of students with IDD in order to plan supports and evaluate their impact on eight vital domains (i.e., emotional wellbeing [EW], material wellbeing [MW], physical wellbeing [PW], personal development [PD], self-determination [SD], social inclusion [SI], interpersonal relationships [IR], and rights [RI]) that are, in turn, sensitive to access, participation, learning, and development of students to their fullest potential (15).

Although QoLSM applications in education are recent, there is a growing recognition of the need to align educational efforts with the implications of this model in order to achieve progress in IE (15–20). One of the main reasons why the QoLSM is useful for promoting IE among these students is because it is aligned with the rights of people with disabilities set out in the UNCRPD (21, 22), as well as with the goals of access, participation, learning, and maximum development set out in the right to IE (17). In this sense, the goal of ‘access’ is aligned with the RI domain; the goal of ‘participation’, is aligned with SI and IR domains; ‘learning’ is aligned with PD and SD; and the goal of ‘development of students to their fullest potential’ is aligned with EW, MW, PW, PD, and SD (15, 23). Thus, applying this model in the educational context can help schools in their task of redefining their cultures, policies, and practices based on a holistic understanding of their students’ aspirations and needs and their consideration in planning and providing support aligned with improving QoL and, by extension, IE-related outcomes. The application of this model in the educational context requires an assessment of individual QoL outcomes, in order to then make decisions based on evidence of personal outcomes, and plan and provide supports aimed at improving the QoL of students in these eight QoL domains linked to the goals of IE. In other words, being able to apply this model in practice in the educational context requires, as a first step, having optimal assessment tools for evaluating QoL in this context.

Consequently, the need arose to develop the Quality of Life Index-Primary Education (QoLI-PE) (15). The QoLI-PE is an assessment tool based on the QoL model, designed to evaluate QoL and provide evidence to inform the planning of supports to promote the personal outcomes of students with IDD enrolled in general primary education in these eight fundamental areas, which are aligned with the aforementioned IE objectives (i.e., access, participation, learning, and maximum possible development) (1). The ultimate goal of this tool is to contribute to student inclusion by identifying priority areas for action, thereby informing the planning and mobilization of necessary supports. Furthermore, the measurement framework provided by this tool allows for an assessment of the results that such support has had on students’ lives, after planning and implementing support over time. Although originally developed for use in Spain (15), the QoLI-PE could also be relevant for fostering an emphasis on QoL and supporting the inclusion of students with IDD in other contexts facing similar challenges, such as students with autism in Chile, where the current research was conducted.

Autism is a developmental condition that manifests itself in early childhood and is characterized by deficits in reciprocal social interaction, social communication, and restricted and repetitive behavior patterns (24). Given the frequent limitations in various activities of daily living, which may or may not coexist with limitations in intellectual functioning, autism is often classified within IDD (11, 25, 26). Although people with autism show great diversity in terms of intellectual and linguistic abilities, the limitations they face can affect their lives in different circumstances, stages of life, and contexts, including the school period and the educational setting (20, 27).

In recent years, the Chilean education system has faced an exponential increase in the enrollment of students with autism in general schools, which has been accompanied by an increase in the prevalence of autism diagnoses worldwide (27–29). While this indicates progress in terms of access to general education in this country, it also creates new demands on an education system that must meet the needs of its students (20, 29, 30). A significant step forward in this regard was the enactment of Chile’s Law No. 21,545 (31), which seeks to guarantee inclusion, including educational inclusion, for people with autism. Specifically, this regulation stipulates that it is the duty of the State to ensure that all individuals with autism receive a high-quality IE and to promote the creation of the necessary conditions for students’ access, participation, retention, and progress—in line with the provisions of the UNCRPD (1). However, for this law to have tangible results in schools, it is essential to provide them with tools and resources that enable them to make evidence-based decisions and monitor the results associated with student inclusion.

For all the above reasons, Chile has prioritized strategies and frameworks for action that are appropriate for meeting the aspirations and needs of students with autism in general education settings, thereby ensuring their access, participation, learning, and development to their full potential. This work stems from the premise that aligning educational efforts with the QoLSM is crucial for promoting the IE of students with autism in general schools in the Chilean context. This is because its measurement and applied implications position it as a useful framework for understanding but also for improving the IE of these students (20). Specifically, the QoLSM allows us, first, to understand the status of these students’ inclusion from a global perspective and in terms of QoL; second, to make evidence-based decisions about the support needed for inclusion; and third, to evaluate the results derived from those supports. Given that the implementation of QoLSM in schools first requires tools for assessing QoL that are appropriate for the context and population, the present study aimed to examine the psychometric properties of the QoLI-PE to assess the QoL of students with autism in Chilean general primary schools, building upon previous research carried out in Spain (15). Thus, this study had the following specific aims: (i) to analyze the evidence of validity regarding the internal structure of the QoLI-PE in a population of students with autism in Chilean general education schools; (ii) to analyze the evidence of reliability regarding the internal consistency of the QoLI-PE in a population of students with autism in general education schools in Chile; and (iii) to explore the QoL status of students with autism in general education schools in Chile.

## 2 Materials and methods

### 2.1 Study design

This study was descriptive, cross-sectional, and instrumental (32, 33), in which the functioning of the QoLI-PE was tested in the Chilean context and the QoL of students with autism in general education schools in this country was explored.

### 2.2 Measures

#### 2.2.1 QoLI-PE Chilean adaptation

The QoLI-PE is a tool focused on the standardized assessment of QoL in students with IDD enrolled in general primary education from the perspective of proxy report, initially developed for the Spanish context. The QoLI-PE addresses a large number of indicators relevant to the QoL of students with IDD in the school setting, in the eight domains mentioned in the introduction (15, 17). This is an online tool that is implemented through a structured interview conducted by an interviewer trained in QoLSM and with knowledge of the instrument, to one or more informants who must have known the student whose QoL is of interest for at least three months and have had recent opportunities to observe them in different situations. The QoLI-PE is structured into seven sections: (i) instructions; (ii) application data; (iii) informant data; (iv) data on the student with IDD being assessed; (v) overall QoL assessment; (vi) QoL assessment scale; and (vii) comments and suggestions. For more details on each section, see the study by Amor et al. (15).

To address the study aims, this study used section vi, “QoL assessment scale.” This section contains 64 items that assess each of the QoL domains included in the Schalock and Verdugo’s model (12): MW, PW, EW, PD, SD, IR, SI, and RI; at a rate of eight items per domain. Each item is answered using a frequency response format: 1 = *Never*, 2 = *Sometimes*, 3 = *Frequently*, and 4 = *Always*. The higher the score, the higher the informant’s rating of the student’s QoL for the domain in question, except for the three reverse items, where the interpretation is the opposite (i.e., 4 = *Never*). The theoretical scores per domain range from 8 (lowest QoL) to 32 (highest QoL).

An adaptation process was conducted to apply the QoLI-PE in the Chilean context. The modifications affected mainly the characterization questions (Section ii to v), especially those associated with the education system (e.g., educational levels, type of school). Except for minor linguistic adjustments, the core content of the QoL assessment items remained unchanged. Thus, the Chilean adaptation retains the same items for assessing students’ QoL across the eight vital domains. In specific instances, certain terms were replaced with local equivalents to ensure cultural and linguistic appropriateness within the Chilean context. However, these modifications do not affect the items’ underlying meaning; therefore, the English translation used in this study is identical to that reported by Amor et al. (15). Details of the items are presented in **Supplementary Table 1** (**Supplementary material****).** Further details of the adaptation process are described in Procedure.

### 2.3 Participants

A total of 164 education professionals completed the QoLI-PE to assess the QoL outcomes of 242 students with autism enrolled in primary education in general schools in four regions of Chile: Tarapacá (57.40%; *n* = 139), Coquimbo (9.10%; *n* = 22), Metropolitan (20.20%; *n* = 49), and Magallanes (13.20%; *n* = 32). Public schools (21.90%; *n* = 53), charter schools (77.30%; *n* = 187), and private schools (0.80%; *n* = 2) participated. All public and subsidized schools had a government-regulated school integration program (99.20%; *n* = 240).

All informants met the inclusion criteria; specifically, they were education professionals who had known the student with autism being assessed for at least three months and had had recent opportunities to observe them in different contexts. Although some informants reported on more than one student, each student’s QoL was assessed by a single informant. Since each informant self-administered the instrument (without an external interviewer), all of them received prior training at the educational center on the QoLSM, the purpose of the project, and how to complete the tool (further details in Procedure). Each informant evaluated an average of 1.48 students (*StDev* = 1.19; *min* = 1; *max* = 10), although it was more common for them to evaluate a single student (*n* = 120 informants), followed by two (n = 29 informants) and, to a lesser extent, three (n = 8 informants), four (n = 5) or ten (n = 2). The informants were mostly female (84.76%). The mean age of the respondents was 36.31 years (*StDev* = 9.04; *min* = 23; *max* = 61) and reported an average of 9.60 years of experience in the field of education (*StDev* = 7.23; *min* = 1; *max* = 40).

As for the students evaluated, they were mostly boys (81.8%) and had an average age of 9.70 years (*StDev* = 2.45; *min* = 6; *max* = 15). Most of the students were born in Chile (97.90%), except for five participants born in Bolivia (2.10%).

The general education system in Chile is organized into three levels: early childhood education, which includes two preschool levels and covers ages 4 and 5; basic education, which covers grades 1 through 8, generally between the ages of 6 and 13, and secondary education, which is generally taken between the ages of 14 and 17 and encompasses four levels between grades 9 and 12. Although grades 1 through 6 correspond in age to what most countries consider primary education, in this case the entire basic education cycle was considered equivalent to primary education, so all levels of this cycle were invited to participate. Thus, the students evaluated in this study were enrolled in basic education levels in the following proportions: first year (*n* = 40; 16.5%), second year (*n* = 42; 17.4%); third year (*n* = 33; 13.6%), fourth year (*n* = 26; 10.7%); fifth year (*n* = 28, 11.6%), sixth year (*n* = 22, 9.2%), seventh year (*n* = 27, 11.2%), eighth year (*n* = 24, 9.9%).

**Table 1** describes informants’ reports regarding the severity of autism of the students assessed and their level of disability according to different criteria, specifically the presence of a diagnosis of intellectual disability (ID), the level of limitations in adaptive behavior across conceptual, social, and practical skills, the reported level of dependence, and the student’s level of support needs. The severity of autism, level of disability, and level of support needs were determined in accordance with the categories outlined in international standards (11, 24), while dependency levels were determined in accordance with the categories set forth in Chilean regulations. Informants reported student data by retrieving information from official educational records and formal assessments. The only exception was the assessment of support needs. In Chile, support needs assessments are not commonly used, so informants reported their assessment based on reference information reported in **Table A1**.

**Table 1:** Severity of autism and level of disability.

Variable	Categories	n (%)
Autism severity	Grade 1	164 (67.80%)
	Grade 2	47 (19.40%)
	Grade 3	13 (5.40%)
	N/A	18 (7.4%)
ID	Autism without ID	202 (83.50%)
	Autism with ID	18 (7.40%)
	Being evaluated for ID	6 (2.50%)
	N/A	16 (6.60%)
Level of disability according to limitations in conceptual skills	Mild	133 (55.00%)
	Moderate	39 (16.10%)
	Severe	18 (7.40%)
	Profound	7 (2.90%)
	No evidence of such limitations	45 (18.60%)
Level of disability according to limitations in social skills	Mild	134 (55.40)%
	Moderate	63 (26.00%)
	Severe	23 (9.50%)
	Profound	9 (3.70%)
	No evidence of such limitations	13 (5.40%)
Level of disability according to limitations in practical skills	Mild	129 (53.30%)
	Moderate	63 (26.00%)
	Severe	14 (5.80%)
	Profound	2 (0.80%)
	No evidence of such limitations	34 (14.00%)
Level of dependency	No recognized dependency	53 (21.90%)
	Mild dependency	125 (51.70%)
	Moderate dependency	43 (17.80%)
	Severe dependency	10 (4.10%)
	N/A	11 (4.50%)
Support needs level	Level I (Intermittent)	136 (56.20%)
	Level II (Limited)	57 (23.60%)
	Level III (Extensive)	33 (13.60%)
	Level IV (Pervasive)	3 (1.20%)
	N/A	13 (5.40%)

N/A, Not available; ID, Intellectual disability.

According to this information, most of the students assessed present low levels of support needs, with the majority having Grade 1 autism severity (n = 164; 67.80%), no ID (n = 202; 83.50%), mild limitations in conceptual (n = 133; 55.00%), social (n = 134; 55.40%), and practical skills (n = 129; 53.30%), mild dependence (n = 53; 21.90%), and low support needs (n = 136; 56.20%).

### 2.4 Procedure

#### 2.4.1 Cultural adaptation process

For the present study, the original QoLI-PE underwent a cultural adaptation process for the Chilean context. The process was led by a team comprising two of the instrument’s original developers (one Spanish and one Chilean) and two researchers from the local study. The adaptation followed international guidelines for the translation and adaptation of psychometric instruments (34–36) to ensure semantic equivalence and contextual appropriateness between the Spanish and adapted versions. This comprehensive review encompassed all seven sections of the tool, rather than focusing exclusively on the QoL assessment scale, and was conducted in three stages: (i) linguistic and contextual appropriateness review and assembly of the preliminary adaptation; (ii) expert evaluation by educational professionals; and (iii) final assembly and validation.

In the first stage, the four aforementioned researchers reviewed the instrument across its various sections to identify necessary terminological or contextual adjustments. Specifically, the two Chilean researchers who were not part of the original study reviewed the instrument to flag elements that were non-functional or unclear within the Chilean context. These observations were then discussed with the original study’s researchers to ensure the conceptual intent of the questions was maintained. Subsequently, the four researchers collaboratively proposed pertinent modifications. During this phase, the involvement of an original study researcher who was also Chilean proved essential, as they possessed a dual understanding of the questions’ original intent and the suitability of the modifications for the Chilean context. Based on this initial review, a preliminary adapted version was drafted, maintaining the QoLI-PE’s original structural composition while integrating contextual and terminological adjustments. Priority was given to **s**emantic equivalence and linguistic naturalness.

In the second stage, this preliminary version was evaluated by external experts. Four educational professionals—a primary school mathematics teacher, a school psychologist, a psychopedagogue, and a special education teacher—participated in this phase. All were currently working in educational centers with students with autism but did not participate in the subsequent study. Each expert was asked to review the instrument and its items for clarity, relevance, and cultural appropriateness. In the third stage, based on feedback from the experts, further adjustments were made in addition to those identified in the previous phases. The result was a final version approved by the four external judges.

The primary modifications were made to the items regarding the characterization of the informant (Section iii) and the student (Section iv). These adjustments addressed specificities of the educational system (e.g., school funding/governance type, informant’s job title) and the national context (e.g., income levels in Chilean pesos instead of euros). A major modification was introduced in Section iv (student characterization) to focus the study specifically on students with autism. While the original version includes a question on the presence of an IDD with various options (e.g., developmental disability without ID)—where autism is an option to be detailed later without prominence—the Chilean version adds a direct question regarding an autism diagnosis (Yes/No). Other modifications throughout the QoLI-PE sections were minor, focusing on replacing terms with more comprehensible alternatives for the Chilean context. Most of these adjustments did not affect the items of the QoL assessment scale. In this section, changes were limited to terms that sounded unnatural in Chilean Spanish. All changes made between the original version and the adaptation are detailed in **Supplementary Table 2** (**Supplementary Material**).

#### 2.4.2 Recruitment procedure

A non-probabilistic convenience sampling strategy was employed through a multi-channel recruitment approach. This included social media outreach, formal invitations to Local Public Education Services (SLEP), and professional networking with school-based colleagues. Additionally, the research team offered complimentary seminars on autism as a proactive engagement tool to motivate institutional participation. The observed regional imbalance in the final sample was primarily due to the fact that professional networking with school-based colleagues—who forwarded the invitations to their respective centers—proved to be the most successful recruitment method.

Following this initial outreach, interested schools received a formal letter detailing the project to confirm their willingness to collaborate. Once institutional participation was formalized, prospective informants from these centers attended a 60-minute training session, conducted either in person or via synchronous online platforms, following a uniform protocol. The training sessions were structured into five key components: (i) an introduction to the QoLSM to align the informants’ conceptual understanding with the study’s objectives; (ii) an explanation of the theoretical underpinnings of the QoLI-PE; (iii) guidance on completing the instrument, including a thorough review of the response scales and specific clarifications regarding the characterization items to minimize error; (iv) a briefing on ethical considerations, emphasizing data confidentiality, anonymity, and the voluntary nature of participation; and (v) a dedicated period for addressing specific questions to ensure full comprehension of the task and the overall study.

Once the training was completed and informed consent was obtained, the Chilean adaptation of the QoLI-PE was administered online using the LimeSurvey platform, hosted on the servers of the Institute for Community Inclusion (INICO) of the University of Salamanca.

The study was reviewed and approved by the Ethics Committee of the Faculty of Social Sciences at the University of Chile. Participation was anonymous, and alphanumeric codes were used to identify both informants and students, thus guaranteeing their anonymity. Authorization was obtained from the various participating centers, and informed consent was obtained from all participants.

### 2.5 Analyses

First, the evidence of validity referring to internal structure and the evidence of reliability referring to internal consistency of the QoLI in its adaptation to the Chilean context were analyzed.

Confirmatory factor analysis (CFA) was used for evidence of validity relating to the internal structure of the instrument, in which the same factor structure reported in the Spanish context was compared (15), consistent in turn with the theoretical model of Schalock and Verdugo (12), that is, an 8–8 model of eight factors (referring to each QoL domain), each consisting of eight items. In addition, it corresponds to an oblique model (i.e., the factors are interrelated). Additionally, a second-order 8-factor model was tested, in which, instead of the factors being correlated, their covariance is explained by a common second-order factor (i.e., QoL).

To test these model, the same items were used as in the study by Amor et al. (15). For the CFA, the WLSMV estimator was used, which is suitable for ordinal variables. The fit was evaluated using the following fit indices: (i) the ratio between Chi-square and degrees of freedom (χ^2^/*df*), adequate if the value was less than 2 (χ^2^/*df* < 2); (ii) the Root Mean Square Error of Approximation (RMSEA) and the Standardized Root Mean Square Residual (SRMR), both with values below.08 indicating an acceptable fit and values below.06 indicating a good fit; and (iii) the Comparative Fit Index (CFI) and the Tucker-Lewis Index (TLI), with values above.90 being adequate and above.95 being optimal (37, 38).

Given that the goal of this study was not to reduce the number of items but rather to produce a Chilean version equivalent to the Spanish version (in order to have comparable data between contexts in the future), unlike the Spanish study, no item cleaning was performed prior to CFA; instead, the same items from the 8–8 model reported in the Spanish study (15) were used. However, the quality of the items in the final solution was analyzed in terms of whether the r squared (*R*^2^) values per item were high (*R*^2^ >.20) and the standardized factor loadings were significant (*p <.050)* and high (*β* >.30) (39).

For reliability evidence, based on factor loadings, internal consistency indices were calculated using ordinal alpha (ordinal α) and omega (ω), which are appropriate for ordinal variables (40).

To characterize the QoL of students with autism attending regular schools in Chile, descriptive analyses were performed considering the items of the QoLI-PE. Given that the model resulting from eight items per QoL domain was used and that raw scores (i.e., the sum of the scores of the individual items, ranging from 1 to 4) were taken as a reference, the observed scores ranged from 8 (lowest level of QoL) to 32 (highest level of QoL) for each domain. The means (*M*), standard deviations (*StDev*), and ranges (minimum-maximum) were calculated for each domain. Additionally, after exploring repeated measures ANOVA with corrections for non-sphericity (Greenhouse-Geisser). Normal Q–Q plots were inspected to evaluate the assumption of normality of the residuals. *Post hoc* analyses were performed to compare QoL domains and determine in which domains students show higher scores at the within-subjects level.

*Post-hoc* analyses were performed using Bonferroni correction (*p*_Bonferroni_) to control the probability of type I error arising from multiple comparisons. For the effect size of the overall contrast, partial eta squared (*η*^2p^) was reported—with values of approximately.01,.06, and.14 indicating small, medium, and large effects, respectively (41)—while for the effect size of the *post-hoc* contrasts, Cohen’s d (*d*) was reported, using Cohen’s interpretation criteria, according to which values close to |.20| indicate a small effect size, values around |.50| indicate a medium effect size, and values close to or greater than |.80| indicate a large effect size (42). All analyses were performed with jamovi 2.3 (43). Findings from the within-subject analysis were contrasted with the non-parametric Friedman test to verify consistency in the conclusions. *Post hoc* testing was performed via Durbin-Conover comparisons, applying a Bonferroni adjustment for multiple testing.

## 3 Results

### 3.1 Validity evidence on the basis of internal structure

**Figure 1:**
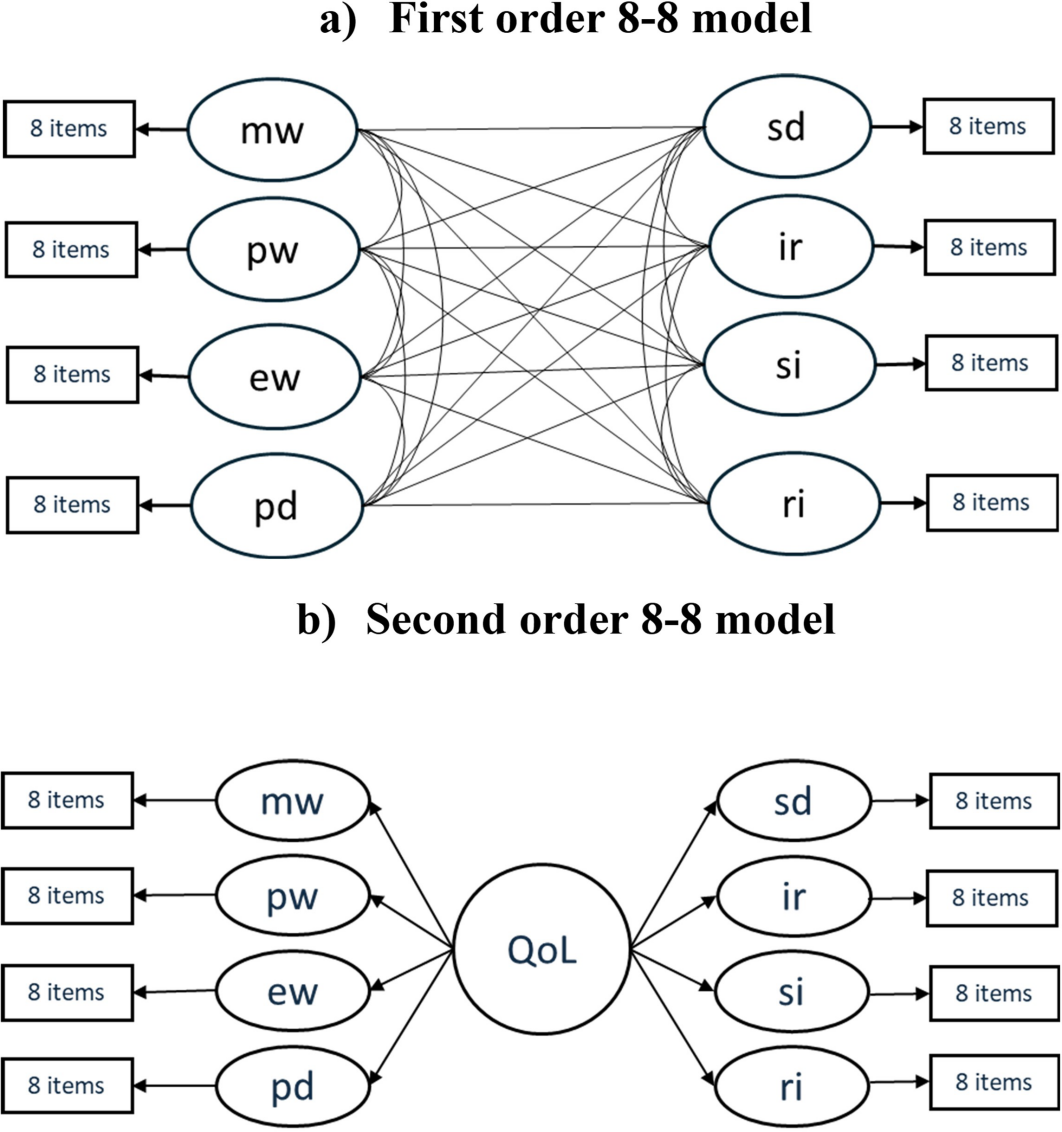
Tested models. QoL, Quality of Life; MW, Material wellbeing; PW, Physical wellbeing; EW, Emotional wellbeing; PD, Personal development; SD, Self-determination; IR, Interpersonal relationships; SI, Social inclusion; RI, Rights. **(a)** First order 8-8 model. **(b)** Second order 8-8 model.

Regarding the fit of the 8–8 model (**Figure 1A**) in the Chilean population, this model demonstrated an optimal fit in all indices (χ²/df = 1.691; RMSEA = .054 [CI = .050 –.057]; CFI = .975; TLI = .974). The model considers 64 items that exhibited significant (*p* <.05) and high standardized factor loadings (all *β* >.40 and most *β* >.50) and high *R*^2^ values (all >.20). All factors showed significant correlations ranging from moderate to high. The highest correlation was found between the IR and SI domains (*r* = .90) and between EW and PD (*r = .*91*)* while the lowest correlation was observed between the MW and IS domains (*r* = .65). **Supplementary Table 3** (**Supplementary material**) details the measurement model, indicating the standardized factor loadings of each item. **Supplementary Table 4** (**Supplementary material**) summarizes the standardized covariances (correlations) between factors (QoL domains).

Given excessive inter-factor correlations were observed between some factors, an alternative structure to that described in Amor et al. (15) was tested, while maintaining theoretical consistency with the QoL model. A second-order eight-factor model (second order 8–8 model) was tested, in which a second-order factor (i.e., overall QoL) explains the common variance among the first-order factors (**Figure 1B**). This solution presented fit indices similar to those of the first-order 8–8 model (χ²/df = 1.719; RMSEA = .055 [CI = .052 –.058]; CFI = .972; TLI = .971), a slightly lower fit, but still optimal according to the above criteria. All items showed significant (*p* <.05) and high standardized factor loadings (all *β* >.40 and most *β* >.50) in relation to each factor, as did each factor in relation to the second-order factor. Both items and factors showed high *R*^2^ values (all items >.20, all factors >.50). **Table 2** details the measurement model, indicating the standardized factor loadings of each item and each factor. As this model avoids the issue of excessively correlated factors, it was deemed more appropriate and parsimonious. Furthermore, it aligns with the QoL model (12), where the eight factors represent distinct domains of a unified, broader construct.

**Table 2:** Second order 8–8 model.

Factor (QoL domain)	Item	β	Factor loading	SE	p	R^2^
MW	mw2	0.616	1.000	0.000	Fixed	0.379
	mw3	0.604	0.980	0.110	<.001	0.364
	mw4	0.578	0.938	0.120	<.001	0.334
	mw5	0.544	0.884	0.132	<.001	0.296
	mw6	0.836	1.358	0.149	<.001	0.699
	mw8	0.650	1.057	0.132	<.001	0.423
	mw9	0.809	1.314	0.143	<.001	0.654
	mw12	0.749	1.217	0.146	<.001	0.562
PW	pw1	0.535	1.000	0.000	Fixed	0.287
	pw2	0.515	0.962	0.093	<.001	0.265
	pw3	0.463	0.865	0.112	<.001	0.215
	pw4	0.782	1.461	0.165	<.001	0.612
	pw7	0.843	1.575	0.165	<.001	0.711
	pw8	0.809	1.511	0.156	<.001	0.655
	pw9	0.616	1.151	0.147	<.001	0.380
	pw10	0.805	1.504	0.145	<.001	0.648
EW	ew2	0.582	1.000	0.000	Fixed	0.339
	ew3	0.582	1.000	0.096	<.001	0.339
	ew4	0.840	1.443	0.134	<.001	0.706
	ew6	0.742	1.274	0.119	<.001	0.550
	ew9	0.630	1.083	0.117	<.001	0.397
	ew10	0.771	1.325	0.128	<.001	0.595
	ew11	0.736	1.264	0.116	<.001	0.541
	ew12	0.807	1.387	0.123	<.001	0.651
PD	pd3	0.631	1.000	0.000	Fixed	0.398
	pd4	0.805	1.276	0.097	<.001	0.648
	pd5	0.668	1.058	0.092	<.001	0.446
	pd6	0.782	1.239	0.095	<.001	0.612
	pd7	0.752	1.191	0.107	<.001	0.565
	pd8	0.828	1.312	0.095	<.001	0.685
	pd9	0.831	1.317	0.093	<.001	0.690
	pd10	0.571	0.905	0.095	<.001	0.326
SD	sd2	0.586	1.000	0.000	Fixed	0.344
	sd3	0.579	0.987	0.103	<.001	0.335
	sd5	0.538	0.917	0.110	<.001	0.289
	sd6	0.612	1.045	0.087	<.001	0.375
	sd7	0.641	1.093	0.101	<.001	0.411
	sd8	0.752	1.283	0.116	<.001	0.566
	sd9	0.832	1.419	0.113	<.001	0.692
	sd11	0.894	1.526	0.122	<.001	0.800
IR	ir1	0.629	1.000	0.000	Fixed	0.396
	ir3	0.618	0.983	0.081	<.001	0.382
	ir4	0.762	1.211	0.091	<.001	0.580
	ir5	0.669	1.063	0.083	<.001	0.447
	ir8*	0.443	0.704	0.082	<.001	0.196
	ir9	0.859	1.365	0.098	<.001	0.738
	ir10	0.778	1.237	0.100	<.001	0.606
	ir11	0.724	1.150	0.098	<.001	0.524
SI	si1	0.742	1.000	0.000	Fixed	0.550
	si2	0.625	0.843	0.068	<.001	0.391
	si3	0.642	0.866	0.072	<.001	0.412
	si4	0.712	0.960	0.062	<.001	0.507
	si5*	0.502	0.677	0.075	<.001	0.252
	si6*	0.484	0.652	0.083	<.001	0.234
	si7	0.702	0.946	0.068	<.001	0.492
	si8	0.927	1.250	0.076	<.001	0.859
RI	ri2	0.677	1.000	0.000	Fixed	0.458
	ri3	0.733	1.083	0.086	<.001	0.537
	ri7	0.739	1.092	0.085	<.001	0.546
	ri8	0.819	1.210	0.094	<.001	0.671
	ri9	0.701	1.036	0.113	<.001	0.491
	ri10	0.864	1.276	0.092	<.001	0.746
	ri11	0.861	1.272	0.104	<.001	0.741
	ri12	0.758	1.121	0.095	<.001	0.575
QoL (second-order factor)	MW	0.805	1.000	0.000	Fixed	0.648
	PW	0.895	0.967	0.121	<.001	0.801
	EW	0.922	1.083	0.138	<.001	0.850
	PD	0.902	1.149	0.144	<.001	0.814
	SD	0.883	1.045	0.140	<.001	0.780
	IR	0.925	1.174	0.151	<.001	0.855
	SI	0.855	1.280	0.155	<.001	0.731
	RI	0.877	1.198	0.152	<.001	0.769

QoL, Quality of Life; β, standardized factor loading; *SE*, Standard error; *R^2^*, r squared; *p*, p value; (*) Denotes reverse item; MW, Material wellbeing; PW, Physical wellbeing; EW, Emotional wellbeing; PD, Personal development; SD, Self-determination; IR, Interpersonal relationships; SI, Social inclusion; RI, Rights. Standardized (β) and non-standardized factor loadings, standard errors of estimation, p and R^2^ values.

Given that some assessments were conducted by the same informant, which could potentially introduce non-independence between observations, the CFA was replicated using a subsample of only one student per informant (n = 164). The results for the second-order 8-subdomain model were consistent with the primary analysis, with the model showing a good fit to the data (χ²/df = 1.932; RMSEA = .052 [CI = .048 –.056]; CFI = .965; TLI = .963), and all items maintained significant and high factor loadings and high *R*^2^ values.

### 3.2 Internal consistency

Reliability was analyzed according to internal consistency for each of the domains, using reliability indices appropriate for ordinal variables (ordinal α and ω). As detailed in **Table 3**, optimal reliability indices (all >.75) were obtained in all domains. Although the second-order model was considered more appropriate, the reliability indices estimated from the model remain the same for both the first- and second-order structures, as they are based on the relationship between each item and its respective factor.

**Table 3:** Indices reporting reliability evidence based on internal consistency.

QoL domain	α	Ω
MW	0.861	0.811
PW	0.859	0.812
EW	0.878	0.841
PD	0.901	0.861
SD	0.874	0.837
IR	0.871	0.838
SI	0.860	0.832
RI	0.914	0.872

QoL, Quality of Life; MW, Material wellbeing; PW, Physical wellbeing; EW, Emotional wellbeing; PD, Personal development; SD, Self-determination; IR, Interpersonal relationships; SI, Social inclusion; RI, Rights; *α=* ordinal Alpha; ω= omega.

### 3.3 QoL outcomes of participating students

**Supplementary Table 1** (**Supplementary Material**) describes the distribution of percentage frequencies obtained for each item in each domain of the QoLI-PE. In both the MW and RI domains, there is a clear concentration of responses at one end of the scale indicating high QoL. In the MW domain, the lowest-rated indicator was item 12 —”*The school is cognitively accessible (i.e., information is provided through different channels, such as auditory, visual, tactile, etc.)*”— which concentrated more than 20% of responses at the pole indicating low QoL. In the RI domain, the lowest-rated indicator was item 7 —”*The school carries out activities to raise awareness of the student’s rights (e.g., murals, games, role-playing, etc.)*”— which had a very different distribution from the rest of the domain, with more than 42% concentrated at the pole indicating low QoL.

In the PW, EW, and PD domains, it can be observed that, although in all items the majority of responses are concentrated at the pole indicating a good QoL, there are some more critical items that indicate areas for improvement. In the PW domain, the most critical item is item 1, which indicates student involvement in physical exercise*—”The student engages in physical exercise according to their personal characteristics”—*in which more than 38% of students concentrated on the low QoL pole. This was followed by item 2*—”The student maintains healthy eating habits (i.e., follows a varied and nutritious diet, drinks an adequate amount of water, etc.)”*—with more than 35% of responses at the negative pole. In the EW domain, the most critical item was item 2 — *“The student feels satisfied with their abilities and skills”* — which concentrated nearly 38% of the ratings in the low QoL pole. In the PD domain, the most critical item was item 10 —*”The projects developed at the school provide opportunities to acquire daily life skills (e.g., learning to use public transport, orienting themselves, cooking, using new technologies, etc.)”*— which concentrated nearly 48% (almost half of the responses) at the low QoL pole.

The SD, IR, and SI domains show a clearly different distribution of responses compared to the other domains, with most of their items concentrating most responses at the indicative pole of low QoL. In SD, the most critical item was item 2 *—”The student seeks alternatives when encountering difficulties in achieving their goals”* — with more than 67% of responses concentrated at the low QoL pole, followed by item 6 *—”The student cooperates with peers to achieve personal and collective goals”*— which accumulated more than 60% in the same direction. In the IR domain, item 1 —*”The student has clearly identified friendships”* — stands out, with more than 46% of responses indicating a low QoL. In the SI domain, the most critical item was item 4 —*”The student’s participation is equitable in group activities”*— with more than 48% of responses indicating low QoL.

In line with what was explored previously, at the descriptive level, the domains with the highest QoL scores were RI (*M =* 27.11; *StDev* = 4.12; *range* = 8-32) and MW (*M* = 27.02; *StDev* = 3.86; *range* = 14-32); followed by EW (M = 26.06; *StDev* = 3.87; *range* = 15-32), PW (M = 25.59; *StDev* = 3.89; *range* = 15-32), PD (*M* = 24.93; *StDev* = 4.25; *range* = 14-32); and with lower scores in SI (*M* = 23.69; *StDev* = 4.74; *range* = 11-32), IR (*M* = 23.70; *StDev* = 4.83; *range* = 8-32) and SD (*M* = 22.71; *StDev* = 4.47; *range* = 11-32), the latter being the lowest.

**Figure 2:**
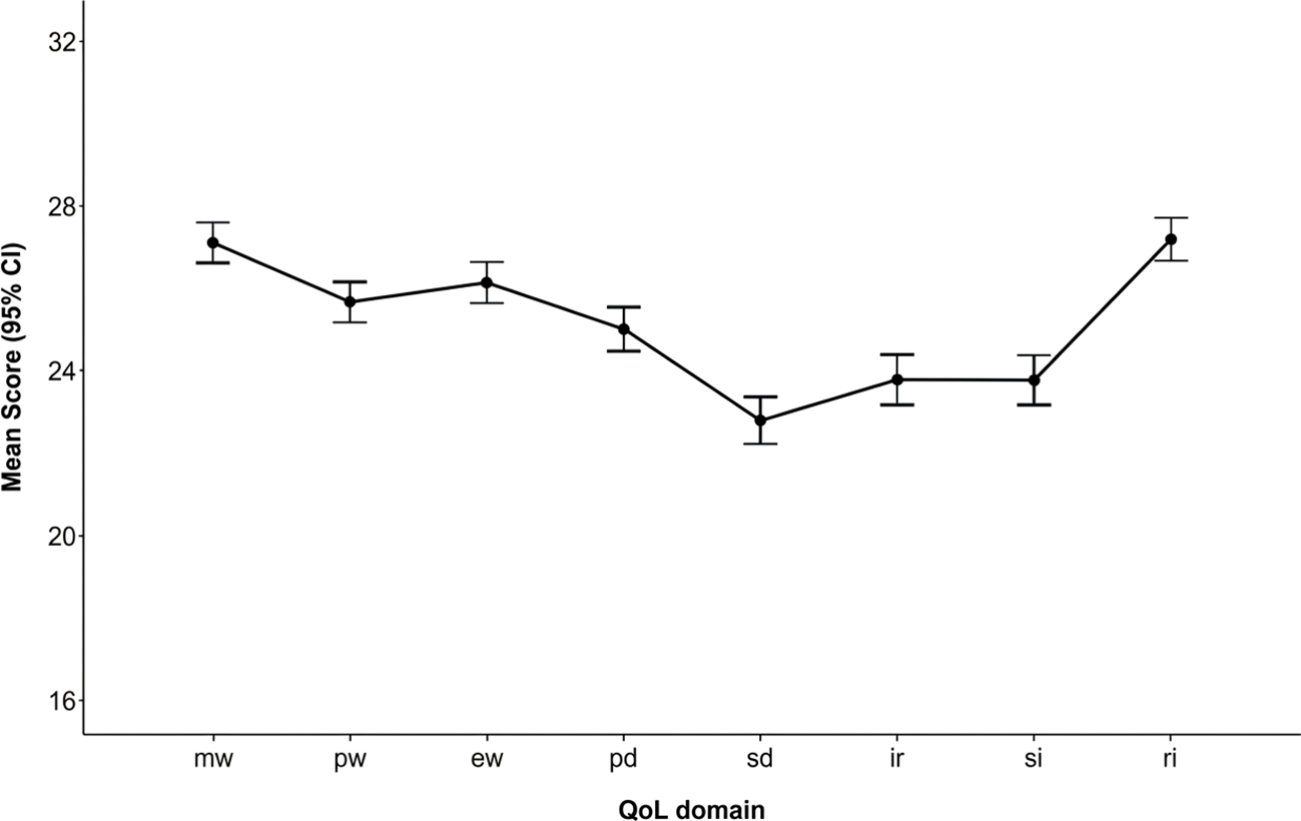
QoL domain means and 95% confidence intervals. Mean QoL domain scores (range 8-32); error bars indicate 95% confidence intervals (N = 242). M, Material wellbeing; PW, Physical wellbeing; EW, Emotional wellbeing; PD, Personal development; SD, Self-determination; IR, Interpersonal relationships; SI, Social inclusion; RI, Rights.

**Figure 2** shows the means and confidence intervals (95%) for each domain. In line with what was reported earlier, this figure shows how the RI and MW domains are rated higher, while the SD, RI, and SI domains emerge as areas of greater concern.

When the means at the within-subjects level were compared using repeated measures ANOVA with sphericity corrections, significant differences were observed between the QoL domains (*F* = 91.5; *p* <.001; *η*^2p^ = 0.275), indicating large effect size. *Post hoc* analyses revealed significant differences between most of the QoL domains. The scores for the two highest-rated QoL domains (RI and MW) were significantly higher than those for the other domains (*p*_Bonferroni_ < 0.001), while they did not differ significantly from each other (*t* = 0.3675; *p*_Bonferroni_ = 1.000; *d* = 0.024).

The EW scores were not significantly higher than those observed in PW (*t* = 2.33; *p*_Bonferroni_= .576; *d* = 0.150) but were higher than those observed in PD (*t* = 6.23; p_Bonferroni_ <.001; *d* = 0.40), IR (*t* = 9.70; *p*_Bonferroni_ <.001; *d* = 0.62), SI (*t* = 8.91; *p*_Bonferroni_ <.001; *d* = 0.57) and SD (*t* = 14.76; *p*_Bonferroni_ <.001; *d* = 0.949). The PW domain was not significantly higher than PD (*t* = 2.97; *p*_Bonferroni_ = .092; *d=* 0.191), but it was significantly higher than IR (*t=* 7.14*; p*_Bonferroni_ <.001; *d =* 0.46), SI (*t =* 7.40*; p*_Bonferroni_ <.001; *d =* 0.48), and SD (*t=* 11.65*; p*_Bonferroni_ <.001; *d =* 0.75). The IR and SI dimensions did not differ significantly from each other (*t* = 0.0197; *p*_Bonferroni_ = 1.000; d ≈ 0.00) and both were significantly higher than the scores reported in SD (*p*_Bonferroni_ <.001).

Given the violation of the normality of residuals and the ordinal nature of the items, analyses were repeated using the non-parametric Friedman test, which also revealed significant differences between domains (χ² = 437; *p* <.001). *Post-hoc* comparisons using Durbin-Conover tests with Bonferroni adjustment yielded consistent trends: MW and RI emerged as the highest-scoring areas with no significant difference between them (*p*_Bonferroni_ = 1.000), while SD showed the lowest scores, differing significantly from all other domains (*p*_Bonferroni_ <.001). Furthermore, SI and IR presented similarly low profiles (*p*_Bonferroni_ = 1.000), scoring lower than all dimensions except SD, thus identifying them as primary areas of concern alongside SD. The only difference in this non-parametric approach was that PW scored significantly higher than PD (*p*_Bonferroni_ = .023), unlike in the previous parametric analysis; however, both remain intermediate areas.

## 4 Discussion

Implementing the QoLSM in educational practice to promote the educational inclusion of students with IDD requires, first and foremost, an adequate measurement of the QoL. An appropriate assessment of QoL in the educational context makes it possible to identify priority areas on which to plan and implement supports aimed at improving personal outcomes linked to access, participation, learning, and maximum development (i.e., goals of IE), while also allowing the monitoring of progress achieved in terms of said goals as a result of these supports. Therefore, in Spain the QoLI-PE began to be developed (15, 17), a standardized tool designed to assess the QoL of students in general education settings, specifically at the primary education level.

The present study focused on students with autism in general education schools in Chile, a population that has experienced an exponential increase in enrollment in recent years, thereby posing significant challenges for educational communities in their efforts to respond to and address these students’ needs. Recent legislation aimed at the inclusion of students with autism in this country entails the adoption of appropriate approaches and measures to make such inclusion feasible and observable. Within this context, applications of the QoLSM appear relevant both to enhance the inclusion of this student population and as a theoretical–applied pathway to support compliance with this legislation. However, its implementation first requires adequate measurement procedures that enable evidence-based decision-making grounded in students’ personal QoL outcomes, as well as the monitoring of progress resulting from implemented actions in order to assess whether the requirements established by the legislation are being met.

How appropriate a tool is may be sensitive to contextual or cultural factors (34). Therefore, when a tool developed in one context is intended to be used in a culturally different context, it is necessary at a minimum to examine and contrast its functioning within the context of interest. Given that the QoLI-PE was originally developed for the Spanish context, the aim of this study was to analyze the functioning of this instrument for the assessment of the QoL of students with autism in general education settings in Chile. This overall aim was addressed through three specific aims: (i) to analyze the evidence of validity regarding the internal structure of the QoLI-PE in a population of students with autism in Chilean general education schools; (ii) to analyze the evidence of reliability regarding the internal consistency of the QoLI-PE in this population and context; and (iii) to explore the QoL status of students with autism in general education schools in Chile.

With regard to the first two aims, the QoLI-PE showed an adequate fit for the 64-item configuration across eight domains (8–8 model), as previously reported in the Spanish context (15), with optimal values observed in both global fit indices and item-level indicators. However, this study reported the fit of both a first-order 8–8 model (with related factors) and a second-order 8–8 model (in which the relationship between factors is explained by a common factor, QoL). This latter model was tested to address the high correlations between factors providing a more parsimonious solution. Likewise, the QoLI-PE demonstrated optimal reliability indices across all domains in the Chilean context. Taken together, these findings provide favorable evidence regarding the measurement properties of the QoLI-PE for assessing the QoL of students with autism in Chile enrolled in general education contexts, although relevant projections discussed later in the text.

Given that the tested first-order 8–8 model indicates that QoL domains are interrelated, this study examined the correlations among domains. Strong associations were observed between IR and SI (which were also identified as critical domains, as discussed later) and between PD and EW. It should be noted that these domains have been clearly distinguished both theoretically and operationally, both in the QoL model (12) and in the various tools derived from it (15, 44–48). In the specific case of the QoLI-PE, the operationalization of each domain was developed in Heras et al. (17). Although the strength in the relationship between IR and SI vary from study to study (i.e., correlations found in the literature range from .33 in Gómez et al. (44) to .69 in Fernández et al. (45), the highest associations have been reported in general education settings (15). Although this might suggest a construct or measurement issue between these two dimensions of the QoLI-PE, we do not believe this to be the case because, while it is to be expected that the indicators for these domains are closely related in school settings, they are theoretically and operationally distinct (17). At schools, this high association is expected given that these two domains appoint to the same IE goal: participation (17, 18). Therefore, this finding would be suggesting that the relationships for peer interaction and the sense of social inclusion go hand in hand in the educational context. A school that implements measures to facilitate the social inclusion of students with IDD is likely to be one that also seeks to foster meaningful interpersonal relationships. In turn, the strong association between PD and EW—which are distinctly different and therefore do not measure the same construct (17)— could be understood from a perspective that often goes unnoticed in schools: receiving opportunities to learn new things and to make progress in daily skills is meaningful for nurturing students’ self-concept, self-efficacy, self-esteem, and emotional well-being (49) (50). Given the close relationships among domains, this study tested a second-order model in which the relationship between the domains is explained by a global factor (i.e., QoL). Although this second-order model was not tested in the Spanish study, it is consistent with both the theoretical model (12) (i.e., 8 domains of QoL) and the original 64-item configuration (15).

The aforementioned findings linked to the psychometric properties of the scores obtained using the QoLI-PE in Chile (i.e., goals i and ii of the present study) have conceptual and applied implications. On the one hand, they provide additional evidence on the theoretical structure of the QoL model (12), a model whose structure has been supported by international research since the beginning of the XXI century in different population groups, like children and adolescents with IDD (46), adults with IDD (47), and people with other conditions not necessarily linked to IDD (48). On the other hand, from a practical perspective, calibrating QoL assessment instruments is a first step towards the application of QoLSM in educational practice in Chile, given that the QoLSM follows a decision-making process always guided by evidence on personal outcomes both as input (i.e., target areas for supports planning) and output measures (i.e., impact of actions taken) (51, 52).

With regard to the third objective, aimed at exploring the QoL status of students with autism in general education settings in Chile, this study made it possible to identify several areas for improvement that require greater support within schools. These needs were observed primarily in the domains of SD, IR, and SI, which were the lowest-rated areas, whereas RI and MW emerged as the highest-rated domains.

A lower rating in the SD domain is not surprising, as it is consistent with what has already been reported in the literature on individuals with IDD using other tools based on the QoL model in related populations, although not specifically in students in general educational settings (53–55), as well as with the findings of studies using the QoLI-PE in primary students with IDD in general education settings (15). Although the findings observed in SD were expected, they provide evidence of the importance of developing school-based opportunities that promote the self-determination of this group from an early age (56, 57).

In contrast, as also found in the present study, the RI domain has been reported as one of the more adequately addressed domains in previous research (15, 54, 58). Amor et al. (15) suggested that higher levels of attainment in the RI domain could potentially be associated with legislative requirements concerning the right to access. However, this trend—as well as the high scores observed in MW—might also reflect alternative factors. These include the specific wording of the scale items, a potential social desirability bias in school reporting, or the inherent ease of assessing tangible aspects over more nuanced, less evident dimensions of QoL. Regardless, genuine inclusion must extend beyond mere access, rights, or material sufficiency; it must address the student across the multiple, interdependent domains that collectively shape their life (1, 17, 18, 59).

In the study by Amor et al. (15) conducted with Spanish students with IDD, the lowest-rated domain after SD was EW, rather than SI and IR as observed in the present study. This difference may be explained by the particular challenges that individuals with autism face in peer relationships and social participation (60, 61), compared with other students with IDD. Indeed, other studies on the QoL of individuals with autism and ID, although not conducted in general educational settings, have reported that individuals with autism show poorer interpersonal relationships and social inclusion than those with ID alone (46). However, it is difficult to make direct comparisons, as these previous studies (46, 61) were not focused on the population of students with autism in general primary education. At the same time, these findings may serve as a warning signal, suggesting that aspects related to these domains may be comparatively neglected, while students with autism in Chilean general education settings may be receiving greater support in other domains. One illustrative example is that nearly half of the respondents reported that their students did not have clearly identified friendships (as indicated in Item 1 of IR) or did not participate equitably in group activities (Item 4 of SI). This highlights the need to provide more explicit supports aimed at improving peer relationships and enhancing opportunities for social participation for students with autism, like peer tutoring or inclusive playgrounds (62).

Some critical items were also identified which, although belonging to non-critical domains, provide indications of the need to offer support in specific aspects of the domain in question. This is particularly relevant when these aspects are sensitive for students with autism. For example, although PW was not identified as a critical domain, Item 9 (*“The lighting and noise conditions of the school are adequate for the student’s needs [i.e., they do not alter their well-being]”)* was rated as reflecting low QoL in 27% of cases. Given the sensory characteristics of students with autism (24), attending to lighting conditions and noise levels in general education settings is necessary to ensure their physical well-being at school.

As another example of a critical item, although the RI domain did not emerge as a critical domain but rather as the highest-rated one, the indicator referring to school-based activities aimed at educating about rights*—”The school carries out activities to raise awareness of the student’s rights (e.g., murals, games, role-playing, etc.)”*—concentrated nearly half of the responses in a category indicative of need for improvement. This suggests that, although RI is a domain that is generally addressed, there is a lack of explicit instruction regarding students’ rights. Such explicit instruction appears to be a fundamental measure in efforts to improve educational inclusion and the protection of students’ rights, and it is also closely related to self-determination, which emerged as a more underdeveloped area.

Among the strengths of this study is the fact that it is the first research conducted in Chile to analyze the IE of students with autism in general education settings from a QoL perspective. In addition, it features a large sample size for a homogeneous population, namely students with autism in general education settings, most of whom attend public schools or charter schools. Another strength is the similarity in the data collection and analysis procedures compared with the Spanish study. Although interviewers were not used in this study (each informant acted as their own interviewer), a training process was implemented for informants, which ensured comparable conditions of administration.

Despite these strengths, this study is not free from limitations. First, the sampling strategy used (i.e., nonprobabilistic) makes it difficult to generalize the results (63). Only some regions of Chile participated in the study, with greater representation from the Tarapacá region. In this regard, future studies should represent the diverse regions of the country. According to data from the Chilean Ministry of Education (64), in 2024 there were over 53,000 students diagnosed with autism enrolled in schools nationwide and over 1,200 school-aged children with an ASD diagnosis were identified in the Tarapacá Region. Consequently, while the current sample represents more than 10% of the student population with ASD in the Tarapacá Region, it accounts for less than 1% of the total national population. This limitation is also related to the characteristics of the sample. The sample includes students who predominantly present low support needs and attend mainly public or charter general education schools. In this sense, the results presented here may not be representative of what would be observed in private schools or among students with higher support needs. Consequently, future studies should consider larger sample sizes to: (i) enhance the representativeness of the study population concerning geographical distribution and students’ varying support needs; and (ii) extend the validation process by establishing national norms and standardizing the instrument for the Chilean context. Since this study has considered only reliability analyses based on internal consistency, future studies could provide other types of reliability evidence, such as test-retest reliability.

Second, this study is limited by its reliance on the perspectives of education professionals, excluding the critical viewpoint of families. This omission is significant because varied reasons: (i) using only professional informants is subject to potential halo effects and institutional desirability bias, which may influence the reported outcomes; (ii) the perceptions of families regarding the QoL of their children are relevant; and (iii) family-based insights might yield divergent conclusions. In fact, a recent study conducted in Spain (65) suggested discrepancies between family members and professionals. In this regard, future studies should incorporate family assessments of the QoL of students with autism in general education settings. To achieve this, it is first necessary to validate the QoLI-PE for family cohorts, as such evidence is currently unavailable even in the Spanish context. Furthermore, to enable robust comparisons between different evaluators, future research should establish measurement invariance across informant types through progressive invariance studies.

Parallel to this limitation, this study did not include the voices of students with autism themselves. The decision was made because, in children aged 6 to 12, communication skills vary greatly, especially in the case of people with autism, who have very different communication support needs (e.g., it would be very difficult to gather data from a self-report perspective from 6-year-old children compared to 12-year-old). Furthermore, this approach is justified by the design of the QoLI-PE itself, which is intended for proxy informants to allow for the assessment of students across a wide range of ages and varying needs, including students with extensive support needs. Therefore, priority was given to having a constant source of information (i.e., the voices of professionals) rather than taking some data from the students themselves and other data from informants. This decision is also consistent with the study in the Spanish context (15). Nevertheless, the voices of students with autism are essential (20). While they were not directly included in this instrumental study, it is crucial for future research to explore the QoL of students with autism from their own perspectives. This could be achieved through adapted methodologies, such as photovoice, which allow for a more inclusive and representative understanding of their lived realities (20). In this regard, a comprehensive approach to the QoL of students with autism should ideally incorporate the students’ own voices, the perspectives of their families, and the insights of educational professionals.

Third, beyond the constraints of informant types, it is important to acknowledge the scarcity of scientific research focused on the application of the QoLSM within general school settings. While the findings have been discussed in light of existing literature, the lack of prior studies limits the possibility of extensive comparative analysis. However, this scarcity simultaneously serves as a core justification for this research. By applying the QoLSM to the educational field, this study addresses a significant gap in literature, providing empirical evidence on how this framework can be operationalized to assess personal outcomes of students with autism in general schools. Additionally, the study does not examine which variables may be associated with or may moderate the observed QoL outcomes. In this regard, future studies could explore in greater depth the factors associated with the QoL of students with autism in general education settings in Chile. To this end, greater variability in students’ characteristics would be desirable, particularly in terms of autism severity or level of disability, for example through the increased inclusion of students with ID or more significant limitations in adaptive behavior.

Finally, a methodological limitation concerns the nested structure of the data, as some informants evaluated more than one student. Specifically, 120 of 164 informants (73.2%) provided assessments for a single student, while the remainder evaluated multiple students, resulting in a low average cluster size. This distribution reflects the practical reality of inclusive schools, where the number of students often exceeds the number of professionals. While this introduces a degree of non-independence in the observations, the impact on parameter estimates is typically negligible when the average cluster size is so close to one (66). Furthermore, multilevel CFA was deemed inappropriate for this study, as such complex models generally require a larger number of multiple-case clusters to ensure model convergence and stable estimates (67). Given these sample characteristics, a standard CFA was the most parsimonious and robust choice. To address this limitation, the AFC was replicated using only the single assessments (164 informants and 164 students), and the same conclusions were reached. Future research could consider evaluating the influence of multiple assessments per informant within a complex model.

Beyond its limitations, this study has important implications for both research and practice. From a research perspective, it provides novel evidence to a growing body of scientific literature on QoL across different countries and contexts, and more specifically contributes new knowledge on the QoL of students with autism in general education settings, with a view to improving their educational inclusion. Moreover, it sets a precedent for future studies that may explore the QoL of this population in greater depth, for example through qualitative or mixed-methods research designs.

From an applied perspective, this study contributes to the availability of an easy-to-administer tool that can support improvements in psychoeducational practices aimed at promoting the school inclusion of students with autism through the enhancement of their QoL (51). The QoLI-PE enables the assessment of students’ personal needs and aspirations, thereby informing the implementation of supports within the educational context from a systems perspective allowing interventions at different levels.

This is possible because the QoLI-PE, like other QoL tools (51), allows evidence-based decisions to be made at different system levels based on evidence about personal QoL outcomes, in this case, at the individual or classroom level (i.e., microsystem), at the school, multi-school, or locality level (i.e., mesosystem), and at the policy level (i.e., macrosystem). At the microstructural level, an assessment using the QoLI-PE would provide insight into the QoL of a given student with autism in a given general school in Chile, identifying needs and aspirations that would guide the planning of support measures aimed at improving their outcomes related to inclusion and evaluating progress in this area. At the mesosystem level, the QoLI-PE allows the use of aggregated data at the school level, which would make it possible to evaluate the extent to which the school’s policies, practices, and cultures address the QoL of students with autism in their inclusion processes. This can help identify barriers that hinder the inclusion of students from an emphasis on their QoL and identify priority areas for improvement at the school level that allow for changes aimed at improving their inclusion. Similarly to what happens at the microsystem level, a new evaluation using the tool would provide evidence of progress or improvements resulting from the actions taken since the initial evaluation. This can also be extrapolated to evaluations at the multi-school level or at the level of localities that have a common management of resources. This is because if different schools that operate in a similar way and are regulated by the same central service present the same critical areas, it would be more efficient to take measures at the regional coordination level or at the local level to address these challenges centrally. In the Chilean context, this could enable a more accurate assessment of school-based initiatives designed to meet the requirements of the new legislation and provide evidence to support the continuous review and improvement of these policies. Finally, at the macrostructure level, the QoLI-PE can provide relevant input for the development, implementation, monitoring, and evaluation of the new autism law in the Chilean context. In this regard, the use of the QoLI-PE would make it possible to provide systematic evidence for monitoring and evaluating compliance with Law No. 21,545 in the educational field, particularly with respect to the State’s duty to ensure inclusive education of quality and to generate conditions for access, participation, retention, and progress of students with autism (Articles 6 and 18), as well as to periodically report on the state of progress in its implementation through observable indicators of quality-of-life outcomes (Article 22) (31).

This study has projections for future research. The main projection is aimed at the possibility of conducting multi-country studies both in terms of measuring QoLI-PE and comparing its results in terms of QoL outcomes for the population of students with IDD in general education contexts. Given that in order to draw conclusions from data obtained from a tool applied in different contexts, it is necessary to provide evidence of invariance between them as a starting point (68, 69). While the adequate fit found in both the Chilean and Spanish contexts suggests a similar underlying structure, it is important to note that the Spanish study did not specifically test a second-order solution as was done in the present work. Regardless of these structural nuances, providing evidence of measurement invariance remains a mandatory prerequisite for drawing valid comparisons between different cultural contexts (61, 62). Therefore, future studies should conduct progressive invariance analyses to determine the extent to which QoLI-PE scores are comparable across countries.

Another projection has to do with consolidating the QoLI-PE as a tool ready for use by Chilean schools that aim to identify the QoL outcomes of their students with autism in terms of their educational inclusion. Although solid evidence of its quality as a measurement tool has already been reported, it is necessary to move forward with the publication of the physical or digital tool, together with an application manual and interpretation scales in a user-friendly format that is easy for schools to use.

In conclusion, the QoLI-PE is a tool with adequate measurement properties that can be useful for improving the inclusion of students with autism in general education contexts in Chile. In the current context and policy environment, it can be used to take evidence-based measures in the different systems involved in the Chilean education system, in line with policy compliance, but above all with improving the inclusion of these students from a rights perspective.

## Data Availability

The raw data used in this study can be found at European Union Open Research Repository ‘Zenodo’: https://doi.org/10.5281/zenodo.18252899.

## Appendix 1

**Table:** Table A1 Description of levels of support needs.

Level	Description
Level I (Intermittent)	Low intensity. Intermittent needs. Requires extraordinary support at specific times, such as when there is an unforeseen event or a change or transition between activities and contexts.
Level II (Limited)	Moderate intensity. Limited needs. Requires ongoing extraordinary support, but the support is not time-consuming.
Level III (Extensive)	High intensity. Extensive needs. Requires extraordinary support on a continuous and regular basis in relation to different environments (not necessarily all) and without time limits.
Level IV (Pervasive)	Very high intensity. Pervasive needs. Requires extraordinary support on a constant and high-intensity basis in different settings, and this support is lifelong.

## References

[1] United Nations . Convention on the rights of persons with disabilities. In: Convention on the rights of persons with disabilities (2006). Available online at: https://www.ohchr.org/en/instruments-mechanisms/instruments/convention-rights-persons-disabilities (Accessed November 5, 2026).

[2] Norwich B Koutsouris G . Addressing dilemmas and tensions in inclusive education. In: Oxford research encyclopedia of education. New York: Oxford University Press (2017). Available online at: http://education.oxfordre.com/view/10.1093/acrefore/9780190264093.001.0001/acrefore-9780190264093-e-154. doi: 10.1093/acrefore/9780190264093.013.154

[3] Alcaraz García S Arnaiz Sánchez P . La escolarización del alumnado con necesidades educativas especiales en España: un estudio longitudinal. Rev Colomb Educ. (2020) (78):299–320. doi: 10.17227/rce.num78-10357

[4] Amor AM Hagiwara M Shogren KA Thompson JR Verdugo MÁ Burke KM . International perspectives and trends in research on inclusive education: a systematic review. Int J incl Educ. (2019) 23:1277–95. doi: 10.1080/13603116.2018.1445304. PMID: 37339054

[5] Buchner T Shevlin M Donovan M Gercke M Goll H Šiška J . Same progress for all? Inclusive education, the United Nations Convention on the Rights of Persons With Disabilities and students with intellectual disability in European countries. J Policy Pract Intellect Disabil. (2021) 18:7–22. doi: 10.1111/jppi.12368. PMID: 40046247

[6] Calderón-Almendros I Echeita-Sarrionandia G . Inclusive education as a human right. In: Oxford research encyclopedia of education. New York: Oxford University Press (2022). Available online at: https://oxfordre.com/education/view/10.1093/acrefore/9780190264093.001.0001/acrefore-9780190264093-e-1243. doi: 10.1093/acrefore/9780190264093.013.1243

[7] Dada S Wilder J May A Klang N Pillay M . A review of interventions for children and youth with severe disabilities in inclusive education. Cogent Educ. (2023) 10:2278359. doi: 10.1080/2331186X.2023.2278359. PMID: 37339054

[8] Grynova M Kalinichenko I . Trends in inclusive education in the USA and Canada. Comp Prof Pedagogy. (2018) 8:28–34. doi: 10.2478/rpp-2018-0016

[9] Villaseca Vial M . Estado de la educación especial en Chile. Santiago of Chile: AcciónEducar. (2023).

[10] Spanish Ministry of Education, Professional Training, and Sports . Non-university teachings. Students enrolled. (2024). Available online at: https://www.educacionfpydeportes.gob.es/servicios-al-ciudadano/estadisticas/no-universitaria/alumnado/matriculado/2023-2024-da.html (Accessed November 10, 2026).

[11] Schalock RL Luckasson R Tassé MJ . Intellectual disability: Definition, diagnosis, classification, and systems of supports. Silver Spring, Maryland: American Association on Intellectual and Developmental Disabilities (2021). 10.1352/1944-7558-126.6.439 34700345

[12] Schalock RL Verdugo MÁ . Handbook on Quality of Life for Human Service Practitioners. Washington, DC, USA: American Association on Mental Retardation (2002).

[13] Gómez LE Schalock RL Verdugo MÁ . A quality of life supports model: Six research-focused steps to evaluate the model and enhance research practices in the field of IDD. Res Dev Disabil. (2021) 119:104112. doi: 10.1016/j.ridd.2021.104112. PMID: 34655955

[14] Verdugo MÁ Schalock RL Gómez LE . The Quality of Life Supports Model as a major component in applying the quality of life paradigm. J Policy Pract Intellect Disabil. (2024) 21:e12468. doi: 10.1111/jppi.12468. PMID: 40046247

[15] Amor AM Sánchez-Gómez V Verdugo MÁ Aza A Wolowiec Z . A new quality of life index to enhance the inclusion of primary education students with intellectual and developmental disabilities in Spain: A preliminary study. Res Dev Disabil. (2025) 161:104975. doi: 10.1016/j.ridd.2025.104975. PMID: 40121712

[16] Gonzalo Ó Heras I Castillo JL Meza C Verdugo MÁ . Impact of the Quality of Life Supports Model on the inclusion of students with disabilities in higher education: A scoping review. Res Dev Disabil. (2024) 154:104850. doi: 10.1016/j.ridd.2024.104850. PMID: 39393332

[17] Heras I Amor AM Verdugo MÁ Calvo MI . Operationalisation of quality of life for students with intellectual and developmental disabilities to improve their inclusion. Res Dev Disabil. (2021) 119:104093. doi: 10.1016/j.ridd.2021.104093. PMID: 34678708

[18] Sánchez-Gómez V López M Amor AM Verdugo MÁ . Apoyos para la Calidad de Vida de Escolares con y sin Discapacidad: Revisión de Literatura. Rev Int Educ Para Justicia Soc. (2020) 9:327–49. doi: 10.15366/riejs2020.9.2.016

[19] Verdugo MÁ Amor AM . Los apoyos y la mejora de la calidad de vida de los alumnos con discapacidad intelectual y del desarrollo. In: Tratamiento paso a paso de los problemas psicológicos en escolares. Madrid: Pirámide (2025). p. 217–34.

[20] Zenteno-Osorio S Sánchez-Gómez V . (Re)pensar la educación desde el bienestar: aplicaciones del modelo de calidad de vida en la inclusión educativa de estudiantes con autismo. In: Construir el Bienestar: Aproximaciones diversas desde las Ciencias Sociales. Iquique: Universidad de Tarapacá (2025).

[21] Gómez LE Morán ML Navas P Verdugo MÁ Schalock RL Lombardi M . Using the quality of life framework to operationalize and assess the CRPD articles and the Sustainable Development Goals. J Policy Pract Intellect Disabil. (2024) 21:e12470. doi: 10.1111/jppi.12470. PMID: 40046247

[22] Lombardi M Vandenbussche H Claes C Schalock RL De Maeyer J Vandevelde S . The concept of quality of life as framework for implementing the UNCRPD. J Policy Pract Intellect Disabil. (2019) 16:180–90. doi: 10.1111/jppi.12279. PMID: 40046247

[23] Turnbull HR Turnbull AP Wehmeyer ML Park J . A quality of life framework for special education outcomes. Remedial Spec Educ. (2003) 24:67–74. doi: 10.1177/07419325030240020201

[24] American Psychiatric Association . Diagnostic and Statistic Manual of Mental Disorders. Washington, DC: American Psychiatric Association (APA) (2022). doi: 10.1176/appi.books.9780890425787.

[25] Liñares-de-Marcos J Palomero-Sierra B Sánchez-Gómez V Fernández-Álvarez CJ Canal-Bedia R . An integrative approach between neurodiversity perspectives and quality of life models for autistic people across the spectrum of support needs. Front Psychiatry. (2026) 16:1756323. doi: 10.3389/fpsyt.2025.1756323. PMID: 41561984 PMC12812635

[26] Tassé MJ Havercamp SM Krahn G Shogren KA Bonardi A Kim M . About whom are we talking when we use intellectual and developmental disabilities? JAMA Pediatr. (2025) 179:83. doi: 10.1001/jamapediatrics.2024.4552. PMID: 39585684

[27] Lord C Charman T Havdahl A Carbone P Anagnostou E Boyd B . The Lancet Commission on the future of care and clinical research in autism. Lancet. (2022) 399:271–334. doi: 10.1016/S0140-6736(21)01541-5. PMID: 34883054

[28] Baio J Wiggins L Christensen DL Maenner MJ Daniels J Warren Z . Prevalence of autism spectrum disorder among children aged 8 years — Autism and developmental disabilities monitoring network, 11 sites, United States, 2014. MMWR Surveill Summ. (2018) 67:1–23. doi: 10.15585/mmwr.ss6706a1. PMID: 29701730 PMC5919599

[29] Núñez-Núñez TP Lagos-Luciano JF Valenzuela-Díaz SO Santander Quiróz GA . Teacher training on evidence-based practices for students with autism spectrum disorder in Chile. Siglo Cero. (2025) 56:65–86. doi: 10.14201/scero.32522

[30] Zenteno-Osorio S Julio Galaz K . Caóticamente hermoso: experiencia de un equipo de aula en el proceso educativo de estudiantes con autismo en una escuela regular del norte de Chile. Rev Enfoques Educ. (2024) 21:123–42. doi: 10.5354/2735-7279.2024.73996

[31] Chilean Ministry of Health . Ley 21545 que Establece la promoción de la inclusión, la atención integral, y la protección de los derechos de las personas con trastorno del espectro autista en el ámbito social, de salud y educación. (2023). Available online at: https://www.bcn.cl/leyChile/navegar?idNorma=1190123 (Accessed December 20, 2026).

[32] Ato M López-García JJ Benavente A . Un sistema de clasificación de los diseños de investigación en psicología. An. Psicol. (2013) 29:1038–59. doi: 10.6018/analesps.29.3.178511

[33] Montero I León O . A guide for naming research studies in Psychology. Int J Clin Health Psychol. (2007) 7:847–62.

[34] International Test Commission (ITC) . The ITC guidelines for translating and adapting test. In: The ITC guidelines for translating and adapting test. Hemel Hempstead: International Test Commission (2017). Available online at: www.intestcom.org (Accessed December 20, 2025).

[35] Beaton DE Bombardier C Guillemin F Ferraz MB . Guidelines for the process of cross-cultural adaptation of self-report measures. Spine. (2000) 25:3186–91. doi: 10.1097/00007632-200012150-00014. PMID: 11124735

[36] Muñiz J Elosua P Hambleton R . Directrices para la traducción y adaptación de los tests: segunda edición. Psicothema. (2013) 25:151–7. doi: 10.7334/psicothema2013.24. PMID: 23628527

[37] Hu L Bentler PM . Cutoff criteria for fit indexes in covariance structure analysis: Conventional criteria versus new alternatives. Struct Equ Model Multidiscip J. (1999) 6:1–55. doi: 10.1080/10705519909540118. PMID: 37339054

[38] Xiao G Zhao Y Huang W Hu L Wang G Luo H . Health economic evaluation of noninvasive prenatal testing and serum screening for down syndrome. PloS One. (2022) 17:e0266718. doi: 10.1371/journal.pone.0266718. PMID: 35421148 PMC9009700

[39] Hair JF Anderson RE Tatham RL Black WC . Análisis multivariante. Madrid: Pearson Prentice Hall (2010).

[40] Domínguez Lara S . Propuesta para el cálculo del Alfa Ordinal y Theta de Armor. Rev Investig En Psicol. (2012) 15:213. doi: 10.15381/rinvp.v15i1.3684

[41] Richardson JTE . Eta squared and partial eta squared as measures of effect size in educational research. Educ Res Rev. (2011) 6:135–47. doi: 10.1016/j.edurev.2010.12.001. PMID: 38826717

[42] Cohen SJ . Measurement of negativity bias in personal narratives using corpus-based emotion dictionaries. J Psycholinguist Res. (2011) 40:119–35. doi: 10.1007/s10936-010-9158-7. PMID: 20972887

[43] The jamovi project . Jamovi. In: Jamovi (2023). Available online at: https://www.jamovi.org (Accessed January 10, 2024).

[44] Gómez LE Alcedo MA Verdugo MA Arias B Fontanil Y Arias VB . KidsLife: Evaluación de la calidad de vida de niños y adolescentes con discapacidad intelectual. Salamanca: INICO. Available online at: https://sid-inico.usal.es/documentacion/escala-kidslife/ (Accessed November 5, 2025).

[45] Fernández M Gómez LE Arias VB Aguayo V Amor AM Andelic N . A new scale for measuring quality of life in acquired brain injury. Qual Life Res. (2019) 28:801–14. doi: 10.1007/s11136-018-2047-5. PMID: 30448910

[46] Arias VB Gómez LE Morán ML Alcedo MÁ Monsalve A Fontanil Y . Does quality of life differ for children with autism spectrum disorder and intellectual disability compared to peers without autism? J Autism Dev Disord. (2018) 48:123–36. doi: 10.1007/s10803-017-3289-8. PMID: 28895015

[47] Verdugo MA Gómez LE Arias B Santamaría M Navallas E Fernández S . San Martín Scale: Quality of life assessment for persons with significant disabilities. Santander: Fundación Obra San Martín (2014).

[48] Aza A Verdugo MÁ Orgaz MB Fernández M Amor AM . Adaptation and validation of the self-report version of the scale for measuring quality of life in people with acquired brain injury (CAVIDACE). Qual Life Res. (2020) 29:1107–21. doi: 10.1007/s11136-019-02386-4. PMID: 31853880

[49] Martínez‐Isla E Morán ML García‐Fernández J Pérez‐Curiel P Vicente E Gómez LE . Personal development and inclusive education in people with intellectual disability: A subscale aligned with the CRPD. J Policy Pract Intellect Disabil. (2026) 23:e70053. doi: 10.1111/jppi.70053. PMID: 40046247

[50] Bendini M Devercelli AE . Quality early learning. Nurturing children's potential. Washington, DC: World Bank Group (2022).

[51] Amor AM Verdugo MÁ Fernández M Aza A Sánchez-Gómez V Wolowiec Z . Development and validation of standardized quality of life measures for persons with IDD. Behav Sci. (2023) 13:452. doi: 10.3390/bs13060452. PMID: 37366704 PMC10295264

[52] European Association of Service Providers for Persons with Disabilities . Quality of Life Index for Inclusive Education and how to use it in the monitoring of the European Child Guarantee. Brussels (2022). Available online at: https://easpd.eu/resources-detail/quality-of-life-index-for-inclusive-education-and-how-to-use-it-in-the-monitoring-of-the-european-child-guarantee/ (Accessed November 5, 2025).

[53] González Martín E Gómez Sánchez LE Alcedo Rodríguez M . Enfermedades raras y discapacidad intelectual: evaluación de la calidad de vida de niños y jóvenes. Siglo Cero Rev Esp Sobre Discapac Intelect. (2016) 47:7. doi: 10.14201/scero2016473727

[54] Morán L Gómez LE Balboni G Monsalve A Verdugo MÁ Rodríguez M . Predictors of individual quality of life in young people with Down syndrome. Rehabil Psychol. (2022) 67:205–14. doi: 10.1037/rep0000443. PMID: 35298204

[55] Morán M Gómez LE Alcedo M . Inclusión social y autodeterminación: los retos en la calidad de vida de los jóvenes con autismo y discapacidad intelectual. Siglo Cero Rev Esp Sobre Discapac Intelect. (2019) 50:29. doi: 10.14201/scero20195032946

[56] Vicente Sánchez E Mumbardó-Adam C Coma Roselló T Verdugo Alonso MÁ Giné Giné C . Autodeterminación en personas con discapacidad intelectual y del desarrollo: revisión del concepto, su importancia y retos emergentes. Rev Esp Discapac. (2018) 6:7–25. doi: 10.5569/2340-5104.06.02.01

[57] Vicente-Sánchez E Guillén-Martín VM Verdugo-Alonso MÁ Calvo-Álvarez MI . El rol de los factores personales y familiares en la autodeterminación de jóvenes con discapacidad intelectual. Psicol Educ. (2018) 24:75–83. doi: 10.5093/psed2018a13

[58] Morán ML Gómez LE Balboni G Bacherini A Monsalve A . Quality of life in children and adolescents with cerebral palsy and intellectual disability: predictors and personal outcomes. Child Indic Res. (2024) 17:123–43. doi: 10.1007/s12187-023-10079-1. PMID: 30311153

[59] Pazey BL Schalock RL Schaller J Burkett J . Incorporating quality of life concepts into educational reform: creating real opportunities for students with disabilities in the 21st century. J Disabil Policy Stud. (2016) 27:96–105. doi: 10.1177/1044207315604364

[60] Guillén VM Verdugo MÁ Jiménez P Aguayo V Amor AM . Support needs of children with autism spectrum disorders: implications for their assessment. Behav Sci. (2023) 13:793. doi: 10.3390/bs13100793. PMID: 37887443 PMC10604162

[61] Morán L Gómez LE Alcedo MÁ . Relaciones interpersonales en niños y jóvenes con trastornos del espectro del autismo y discapacidad intelectual. Rev Esp Discapac. (2015) 3:77–91. doi: 10.5569/2340-5104.03.01.04

[62] Unidad especializada de orientación del ámbito de discapacidad intelectual de Játiva . Construir una escuela donde todos y todas sumamos. Día internacional de las personas con discapacidad. Játiva (2023) (Accessed November 5, 2025).

[63] Boyd RJ Powney GD Pescott OL . We need to talk about nonprobability samples. Trends Ecol Evol. (2023) 38:521–31. doi: 10.1016/j.tree.2023.01.001. PMID: 36775795

[64] Chilean Ministry of Education . Matrícula Oficial 2013-2025. Santiago: Chilean Ministry of Education. (Accessed February 12, 2026).

[65] García López I Sánchez-Gómez V Fernández Sánchez M Guillén Martín V Alvarado Torres N . Inclusión del alumnado de primaria con discapacidades intelectuales y del desarrollo: percepciones de familiares y profesionales desde un enfoque de calidad de vida. Siglo Cero. (In press).

[66] Muthen BO Satorra A . Complex sample data in structural equation modeling. Sociol Methodol. (1995) 25:267. doi: 10.2307/271070. PMID: 34318309

[67] Maas CJM Hox JJ . Sufficient sample sizes for multilevel modeling. Methodology. (2005) 1:86–92. doi: 10.1027/1614-2241.1.3.86

[68] Fischer R Karl JA . A primer to (cross-cultural) multi-group invariance testing possibilities in R. Front Psychol. (2019) 10:1507. doi: 10.3389/fpsyg.2019.01507. PMID: 31379641 PMC6657455

[69] Jang S Kim ES Cao C Allen TD Cooper CL Lapierre LM . Measurement invariance of the satisfaction with life scale across 26 countries. J Cross-Cult Psychol. (2017) 48:560–76. doi: 10.1177/0022022117697844

