# Supplementary material for: Measuring the quality of life of students with autism in Chilean general education schools
Source: Front Psychiatry. 2026 May 20;17:1790139. doi: 10.3389/fpsyt.2026.1790139 (PMC13230136; doi:10.3389/fpsyt.2026.1790139)
Supplement: Supplementary file 1 [file Table1.pdf]

Table S1.
Response distribution by item

Item (translated from Spanish)	Response options distribution (%)			
	Never	Sometimes	Frequently	Always
MW2. The student has the necessary materials to carry out school activities (e.g., books, notebooks, reading and writing materials, sports equipment, etc.)	1.24%	6.20%	27.27%	65.29%
MW3. Their materials are replenished or repaired when worn out or damaged	1.65%	13.64%	38.43%	46.28%
MW4. The student has access to a loan service for materials (e.g., laptops, language dictionaries, drawing materials, books, etc.) that meet their educational needs	2.07%	8.26%	35.95%	53.72%
MW5. The necessary resources are provided for them to attend activities organized by the center (e.g., financial aid, adapted transport, reference person, etc.)	7.44%	9.92%	29.34%	53.31%
MW6. The people in their support circle are informed about the different resources (e.g., social, educational, and health) available in the community	0.41%	6.20%	32.64%	60.74%
MW8. The school has the necessary support materials to ensure communication for all students (e.g., computer applications, augmentative and alternative communication systems, etc.)	4.13%	15.70%	34.30%	45.87%
MW9. There is effective communication between the educational team and the families to inform them of the supports needed	0.00%	7.02%	29.75%	63.22%
MW12. The school is cognitively accessible (i.e., information is provided through different channels, such as auditory, visual, tactile, etc.)	2.48%	19.83%	38.02%	39.67%
PW1. The student engages in physical exercise according to their personal characteristics	2.89%	35.95%	32.23%	28.93%
PW2. The student maintains healthy eating habits (i.e., follows a varied and nutritious diet, drinks an adequate amount of water, etc.)	7.02%	29.34%	44.21%	19.42%
PW3. The student maintains healthy hygiene and personal care conditions	1.24%	16.12%	36.36%	46.28%
PW4. Their physiological needs (i.e., the need to rest, go to the bathroom, move, etc.) are respected in the educational dynamics	0.00%	5.37%	21.90%	72.73%
PW7. The teaching staff and support personnel know how to respond to the student's health needs	0.00%	8.68%	38.43%	52.89%
PW8. The conditions of the school guarantee the student’s safety (e.g., specific measures are taken to avoid risks such as falls or blows)	0.00%	11.98%	30.58%	57.44%
PW9. The lighting and noise conditions of the school are adequate for the student’s needs (i.e., they do not alter their well-being)	2.89%	24.79%	47.11%	25.21%
PW10. The educational community promotes the student’s participation in activities that foster the acquisition of healthy habits (e.g., sleep, nutrition, and physical activity)	2.07%	18.60%	40.08%	39.26%
EW2. The student feels satisfied with their abilities and skills	1.65%	36.36%	45.45%	16.53%
EW3. The student enjoys the time spent outside the classroom (e.g., recess, cafeteria, transport, etc.)	0.83%	12.81%	43.80%	42.56%
EW4. The student receives support from people in the educational community (e.g., teachers, family members, students, etc.) when they are in a situation that causes them discomfort	0.00%	3.72%	21.49%	74.79%
EW6. People in the educational environment identify observable behaviors that express the student’s emotional states (e.g., gestures of pleasure, facial expressions of anger, postural expressions of discomfort, etc.)	0.83%	14.46%	37.19%	47.52%

EW9. The educational team records relevant aspects such as the student's behavior, motivations, needs, or interests (e.g., through a personal file)	3.72%	21.07%	33.88%	41.32%
EW10. The student's needs are assessed at the school (e.g., emotional, social, academic, etc.) with the aim of providing supports that promote their well-being	0.00%	9.50%	40.08%	50.41%
EW11. Socio-emotional competencies (e.g., emotional awareness, emotional regulation, emotional autonomy, social awareness) are worked on in the educational dynamics	1.65%	19.83%	43.80%	34.71%
EW12. Specific measures are taken to prevent disruptive behaviors (e.g., self-harm, violent behavior towards others, etc.)	0.41%	13.64%	38.43%	47.52%
PD3. The student has supports aimed at organization and time planning (e.g., agendas, flexible schedules, etc.)	4.13%	27.27%	40.91%	27.69%
PD4. The student has opportunities to develop self-awareness	0.41%	21.90%	44.63%	33.06%
PD5. The people in the student's support circle receive guidance on how to enhance the student's personal development	2.07%	19.01%	52.07%	26.86%
PD6. The educational action is built on the knowledge and skills the student already has	0.00%	11.98%	46.28%	41.74%
PD7. The educational team has positive expectations about the student's potential to acquire and develop competencies (e.g., social, emotional, musical, linguistic, mathematical, physical, etc.) in the school	0.41%	6.61%	31.82%	61.16%
PD8. The educational team uses methodologies that favor the student's learning and motivation (e.g., collaborative work, peer tutoring, corner pedagogies, project work, etc.)	0.41%	15.70%	42.15%	41.74%
PD9. The school creates conditions for the student to practice their competencies (i.e., social, emotional, musical, linguistic, mathematical, physical, etc.)	0.83%	19.01%	43.39%	36.78%
PD10. The projects developed at the school provide opportunities to acquire daily life skills (e.g., learning to use public transport, orienting themselves, cooking, using new technologies, etc.)	7.85%	40.91%	32.23%	19.01%
SD2. The student seeks alternatives when encountering difficulties in achieving their goals	8.26%	59.09%	26.86%	5.79%
SD3. The student communicates their personal needs (e.g., if they need to go to the bathroom, if they are hungry, etc.)	2.48%	18.60%	33.06%	45.87%
SD5. The student expresses which activities they like and which they do not	2.89%	30.58%	27.27%	39.26%
SD6. The student cooperates with peers to achieve personal and collective goals	10.33%	51.24%	24.79%	13.64%
SD7. The student chooses the activities they do in their free time (e.g., recess, after school, weekends, etc.) based on their preferences and desires	3.72%	27.27%	40.08%	28.93%
SD8. The people in the student's support circle receive guidance on how to promote the student's self-determination (e.g., how to facilitate the development of their autonomy, how to enhance their ability to choose and control, etc.)	2.07%	26.03%	47.93%	23.97%
SD9. The teaching staff takes the student's opinions and preferences into account when designing various classroom and school activities	3.31%	29.75%	36.36%	30.58%
SD11. The methodology used provides opportunities to practice skills such as decision-making and problem-solving	2.89%	26.03%	47.11%	23.97%
IR1. The student has clearly identified friendships	14.46%	32.23%	23.14%	30.17%
IR3. The student has trusting relationships with teachers and support staff (e.g., can share their problems, concerns, successes, etc.)	7.85%	25.62%	33.88%	32.64%
IR4. The student has opportunities to work in teams with peers (with and without Specific Educational Support Needs)	4.55%	18.60%	29.34%	47.52%
IR5. The student participates in projects that promote cooperation and mutual help among students (e.g., peer tutoring, mentoring programs, etc.)	16.53%	35.12%	26.03%	22.31%
IR8. The student faces barriers to relating to others **(*)**	14.88%	55.37%	20.25%	9.50%

IR9. The educational team promotes the learning of various social skills (e.g., active listening, courtesy, assertiveness, and respect)	0.00%	10.33%	38.02%	51.65%
IR10. The educational community proposes activities that facilitate maintaining and increasing the student’s social network	3.31%	26.86%	39.26%	30.58%
IR11. People in the educational community use a communication system adapted to the student’s needs (e.g., use accessible language, use visual aids, etc.)	4.96%	14.05%	35.54%	45.45%
SI1. The student participates in inclusive community activities (e.g., leisure activities, cultural activities, etc.)	7.44%	40.08%	26.86%	25.62%
SI2. The student participates in outings organized by the school (e.g., excursions, end-of-year trips, etc.)	10.33%	20.66%	26.03%	42.98%
SI3. The student feels a sense of belonging to their group/class	6.20%	29.34%	31.82%	32.64%
SI4. The student’s participation is equitable in group activities	10.33%	38.43%	26.45%	24.79%
SI5. The student faces barriers that hinder their participation in classroom and educational center activities **(*)**	23.55%	58.26%	14.46%	3.72%
SI6. The student faces barriers to accessing and participating in various community services (e.g., transportation, libraries, civic centers, sports centers, swimming pools, etc.) **(*)**	47.52%	41.74%	7.85%	2.89%
SI7. The student’s participation in inclusive games during recess is promoted (e.g., through dynamic playgrounds)	11.98%	33.47%	24.79%	29.75%
SI8. The teaching staff and support personnel promote the student’s active participation in the development of various activities	0.41%	14.05%	38.02%	47.52%
RI2. The student has the necessary supports to exercise and defend their rights	1.24%	22.31%	28.93%	47.52%
R13. The student can express themselves freely in the educational environment, without violating the rights of others	1.65%	13.22%	28.10%	57.02%
RI7. The school carries out activities to raise awareness of the student’s rights (e.g., murals, games, role-playing, etc.)	7.44%	35.12%	33.06%	24.38%
RI8. The people in the educational community develop specific actions to prevent situations of violence (verbal, physical, and psychological)	0.41%	16.53%	28.93%	54.13%
RI9. The people in the educational community treat the student with respect.	0.41%	2.89%	12.81%	83.88%
RI10. The people in the educational community know how to defend the students’ rights	0.41%	8.26%	26.86%	64.46%
RI11. The school has mechanisms to ensure the student’s protection (e.g., protocols against bullying, family or community violence, child abuse, etc.)	0.41%	4.96%	19.83%	74.79%
RI12. The school develops specific measures to ensure that both the student assessed, and their relatives and support circle can be part of the decision-making process regarding the student’s educational process	2.07%	12.40%	36.78%	48.76%

Note. MW = Material wellbeing; PW = Physical wellbeing; EW = Emotional wellbeing; PD = Personal development; SD = Self-determination; IR = Interpersonal relationships; SI = Social inclusion; RI = Rights; * Denotes reverse items for which response options score the opposite as for straight items. Items with variability problems identified at this stage are highlighted in bold font.
