# Supplementary material for: Measuring the quality of life of students with autism in Chilean general education schools
Source: Front Psychiatry. 2026 May 20;17:1790139. doi: 10.3389/fpsyt.2026.1790139 (PMC13230136; doi:10.3389/fpsyt.2026.1790139)
Supplement: Supplementary file 2 [file Table2.pdf]

Table S2.

Changes made from the original version to the Chilean version of the QoLI-PE

Section	Spanish version	Chilean version	Explanation of the change
**iii. Informant data**	*"NIF/NIE persona informante"*	*"RUT/ RUN persona informante"*	The Spanish identification number NIF/NIE is replaced by the Chilean equivalent RUT/RUN, bringing the instrument into line with the national identity registration system. This information was optional and was requested only if participants wished to receive a certificate of participation.
**iii. Informant data**	*"Tipo de centro que trabaja actualmente:<br>-Centro (ordinario) de Educación Primaria.<br>-Centro de Educación Especial.<br>-Entidad que presta apoyos al alumnado en centros ordinarios.<br>-Gabinete psicopedagógico escolar."*	*"Tipo de centro en el que trabaja actualmente:<br>-Establecimiento educacional regular sin Programa de Integración Escolar (PIE).<br>-Establecimiento educacional regular con Programa de Integración Escolar (PIE).<br>- Escuela Especial."*	This question refers to the type of school where the informant works. The response options have been adapted to the structure of the Chilean education system.
**iii. Informant data**	*"Relación con el/la estudiante evaluado/a:<br>-Maestro/a no tutor.<br>-Tutor/a de su grupo aula.<br>-Orientador/a.<br>-Asistente técnico educativo.<br>-Especialista de Audición y Lenguaje.<br>-Especialista de Pedagogía Terapéutica.<br>-Profesor/a Técnico de Servicios a la Comunidad.<br>-Persona de apoyo de una organización externa.<br>-Otra."*	*"Relación con el/la estudiante evaluado/a:<br>-Profesor/a asignatura.<br>-Profesor/a tutor/a.<br>-Orientador/a.<br>-Técnico de aula.<br>-Fonoaudiólogo/a.<br>-Profesor/a de educación especial.<br>-Trabajador/a social.<br>- Persona de apoyo de una organización externa.<br>-Psicólogo/a<br>-Otra"*	The original response options regarding the role of the informant have been modified in accordance with the classification and regulations of the Chilean education system

**iv. Data on the student with IDD being assessed**	*"Años que lleva el/la estudiante viviendo en España."*	*"Años que lleva el/la estudiante viviendo en Chile."*	This question asks how many years the student has been living in the country, if they were not born there. The geographical reference is adjusted to the country where the survey is being administered.
**iv. Data on the student with IDD being assessed**	There was no question regarding belonging to an indigenous people.	*"El/la estudiante ¿pertenece a algún pueblo originario?*<br>*Sí*<br>*No*<br>*-NS/NC. Falta información."*	A question has been added to recognize the ethnic and cultural diversity of Chilean students, in accordance with the principle of interculturalism established in Chilean education legislation.
**iv. Data on the student with IDD being assessed**	*"Nivel adquisitivo de la familia (en euros):*<br>*-Hasta 500.*<br>*-Entre 500 y 1000.*<br>*-Entre 1000 y 1500.*<br>*-Entre 1500 y 2000.*<br>*-2000 o más*<br>*NS/NC Falta información"*	*"Nivel adquisitivo de la familia (en pesos chilenos):*<br>*-Hasta 500.000*<br>*-Entre 500 y 1.000.000.*<br>*-Entre 1.000.000 y 1.500.000.*<br>*-Entre 1500.000 y 2000.000.*<br>*-2.000.000. o más*<br>*NS/NC Falta informació*n"	In the question regarding household income, the currency unit is changed from euros to Chilean pesos
**iv. Data on the student with IDD being assessed**	*"Localidad y provincia en la que reside."*	*"Comuna en la que reside*."	In the question regarding place of residence, the categories "*localidad*" and "*provincia*" wew replaced with "*comuna*" to align the item with the current political-administrative division in Chile, where the "*comuna*" constitutes the basic territorial unit recognized by the Chilean State.
**iv. Data on the student with IDD being assessed**	*"Titularidad del centro:*<br>*-Pública.*<br>*-Privada.*<br>*-Concertada."*	*"Dependencia del centro educativo:*<br>*-Pública (Municipal/SLEP).*<br>*-Privada.*<br>*-Particular subvencionada."*	This question referred to the type of funding for the student's school. In the adaptation, the term "ownership" has been replaced with "affiliation" to align with local terminology. Additionally, the options have been modified to reflect the types of funding outlined in Chilean legislation.
**iv. Data on the student with IDD being assessed**	*"Lengua:*<br>*-Castellano.*<br>*-Euskera.*<br>*-Catalán.*<br>*-Gallego.*<br>*-Otra."*	*"Lengua:*<br>*-Español/Castellano.*<br>*-Inglés.*<br>*Otra.*<br>*Otra."*	This question refers to the student's native language. The original version lists Spain's various official languages, including the option "Other." While there are several indigenous languages in Chile, they are quite diverse and rarely serve as a student's primary native language. For this reason, it has been determined that such languages can be categorized under the "Other" option.

**iv. Data on the student with IDD being assessed**	*"Modalidad de escolarización:*<br>*-Centro ordinario.*<br>*-Centro Ordinario de Atención Educativa Preferente.*<br>*-Escolarización combinada.*<br>*-Aula Sustitutoria de Centro de Educación Especial.*<br>*-Centro de Educación Especial."*	*"Modalidad de escolarización:*<br>*-Establecimiento educacional regular sin Programa de Integración Escolar (PIE).*<br>*-Establecimiento educacional regular con Programa de Integración Escolar (PIE).*<br>*- Escuela Especial."*	This question refers to the student's educational modality. The answer choices have been modified to align with the structure of the Chilean education system.
**iv. Data on the student with IDD being assessed**	*"Curso que está realizando actualmente.*<br>*-Primero.*<br>*-Segundo.*<br>*-Tercero.*<br>*-Cuarto.*<br>*-Quinto.*<br>*-Sexto."*	*"Curso que está realizando actualmente.*<br>*-Primero.*<br>*-Segundo.*<br>*-Tercero.*<br>*-Cuarto.*<br>*-Quinto.*<br>*-Sexto.*<br>*-Séptimo.*<br>*-Octavo."*	This question refers to the grade level the student is currently in. The original response options, which correspond to the Spanish primary education system, have been adjusted to align with the levels recognized in Chile within basic education ("*Educación básica").*
**iv. Data on the student with IDD being assessed**	*"Indique si la persona evaluada tiene discapacidad intelectual u otra discapacidad del desarrollo:*<br>*Sí*<br>*No"*	*"El/la estudiante evaluado/a ¿Presenta diagnóstico de trastorno del espectro autista (TEA)?*<br>*Sí*<br>*No*<br>*NS/NC. Falta información"*	A specific alternative question is asked first to check for autism; answering "Yes" leads to the same path as answering 'Yes' in the original version, after answering the question about the severity of the autism (detailed below). To ensure the tool remains useful for cases of developmental disabilities other than autism, if you answer "No" to this question, the original question appears next.
**iv. Data on the student with IDD being assessed**	No question on autism severity was included in the original version.	*"En caso de tener un diagnóstico de trastorno del espectro autista (TEA) indique nivel de severidad conocido*<br>*-Grado 1. Necesita ayuda.*<br>*-Grado 2. Necesita ayuda notable.*<br>*-Grado 3. Necesita ayuda muy notable.*<br>*-NS/NC. Falta información"*	A question is added asking about the severity of autism according to the DSM-5, if the respondent answered "yes" to the specific question about autism.

**iv. Data on the student with IDD being assessed**	*"Indique si la persona tiene una discapacidad intelectual u otra discapacidad del desarrollo:*<br>*-Tiene discapacidad intelectual*<br>*-Tiene otra discapacidad del desarrollo CON discapacidad intelectual*<br>*-Tiene otra discapacidad del desarrollo SIN discapacidad intelectual*"	*"Indique si el/la estudiante tiene:*<br>*-Tiene un diagnóstico de TEA y un diagnóstico de discapacidad intelectual*<br>*-Tiene un diagnóstico de TEA sin diagnóstico de discapacidad intelectual.*<br>*-Está en evaluación.*<br>*-NS/NC. Falta información"*	A specific alternative question has been added. If the student was identified as having autism in the previous question, this question asks whether that condition coexists with a diagnosis of intellectual disability or whether the student is currently being evaluated for one. If no diagnosis of autism was reported, respondents will see the original question, which asks whether the student has an intellectual disability, another developmental disability with intellectual disability, or a developmental disability without intellectual disability.
**iv. Data on the student with IDD being assessed**	*"Nivel de dependencia reconocido:*<br>*-Grado I (moderada)*<br>*-Grado II (severa)*<br>*-Grado III (gran dependencia)*<br>*-NS/NC. Falta información.*<br>*-No tiene dependencia."*	*"Nivel de dependencia reconocido:*<br>*-Dependencia Leve.*<br>*-Dependencia moderada.*<br>*-Dependencia severa.*<br>*-NS/NC. Falta de información.*<br>*-No tiene dependencia."*	The original response options referring to dependency levels (I, II, III) are replaced by the categories "leve, moderada, severa," in line with the official classification used by the National Disability Service (SENADIS) in Chile.
**iv. Data on the student with IDD being assessed**	"*Otras condiciones de la persona evaluada"*<br>The original question lists a diverse conditions, including autism.	*"Otras condiciones de la persona evaluada"*<br>Autism was removed from the list of options since it had already been addressed in the previous question; instead, sensory and digestive issues were added.	This question listed a series of conditions in addition to IDD that the student might have. The original list included autism as an additional option. In the Chilean adaptation, this option was removed here, as it had already been addressed by the previous alternative question. Sensory and digestive issues were added under "other conditions," as they are common in autism.
**iv. Data on the student with IDD being assessed**	The original version did not include a question regarding whether the student was aware of their autism diagnosis	"*El o la estudiante, ¿sabe que tiene un diagnóstico de TEA?*<br>*-El/la estudiante sabe que tiene un diagnóstico de TEA.*<br>*-El/la estudiante no sabe que tiene un diagnóstico de TEA."*	A question has been added to explore whether students are aware of their autism diagnoses.
**vi. QoL assessment scale**	Item MW4. The term "*portátil*" is used.	Item MW4. The term "*portátil*" was replaced with "*computador portátil*".	The vocabulary is adapted to the common usage of Chilean Spanish to ensure that the informants can clearly understand it.

**vi. QoL assessment scale**	Item SI2 includes the examples *"excursiones, viajes de fin de curso"*.	Item SI2. The examples includes one more option: *"excursiones, viajes de fin de curso, salidas pedagógicas"*.	The example is adapted to the Chilean school practice.
**vi. QoL assessment scale**	Item SI7 includes the example *"patios dinámicos"*.	Item SI7. The examples includes one more option: "*patios dinámicos o recreos dirigidos*"	The example is adapted to the Chilean school practice.
