# Supplementary material for: Measuring the quality of life of students with autism in Chilean general education schools
Source: Front Psychiatry. 2026 May 20;17:1790139. doi: 10.3389/fpsyt.2026.1790139 (PMC13230136; doi:10.3389/fpsyt.2026.1790139)
Supplement: Supplementary file 3 [file Table3.pdf]

Table S3.

First order 8-8 model. Standardized (β) and non-standardized factor loadings, standard errors of estimation, p and R^2 values.

Factor (QoL domain)	Item	*β*	*Factor loading*	SE	*p*	R^2
MW	mw2	0.617	1.000	0.000	Fixed	0.381
	mw3	0.602	0.975	0.109	<.001	0.363
	mw4	0.578	0.936	0.119	<.001	0.334
	mw5	0.544	0.881	0.131	<.001	0.296
	mw6	0.836	1.353	0.147	<.001	0.698
	mw8	0.652	1.056	0.130	<.001	0.425
	mw9	0.808	1.309	0.142	<.001	0.653
	mw12	0.749	1.213	0.144	<.001	0.561
PW	pw1	0.534	1.000	0.000	Fixed	0.285
	pw2	0.516	0.966	0.094	<.001	0.266
	pw3	0.464	0.869	0.112	<.001	0.215
	pw4	0.781	1.463	0.165	<.001	0.611
	pw7	0.843	1.578	0.166	<.001	0.710
	pw8	0.808	1.513	0.157	<.001	0.653
	pw9	0.619	1.160	0.148	<.001	0.383
	pw10	0.806	1.509	0.146	<.001	0.650
EW	ew2	0.581	1.000	0.000	Fixed	0.338
	ew3	0.581	1.000	0.096	<.001	0.338
	ew4	0.840	1.444	0.135	<.001	0.705
	ew6	0.742	1.276	0.120	<.001	0.550
	ew9	0.631	1.085	0.117	<.001	0.398
	ew10	0.772	1.328	0.129	<.001	0.596
	ew11	0.736	1.266	0.117	<.001	0.542
	ew12	0.807	1.387	0.124	<.001	0.651
PD	pd3	0.631	1.000	0.000	Fixed	0.398
	pd4	0.804	1.275	0.097	<.001	0.646
	pd5	0.666	1.057	0.092	<.001	0.444
	pd6	0.782	1.240	0.095	<.001	0.611
	pd7	0.753	1.194	0.107	<.001	0.567
	pd8	0.829	1.314	0.095	<.001	0.687
	pd9	0.832	1.319	0.093	<.001	0.692
	pd10	0.573	0.909	0.095	<.001	0.328
SD	sd2	0.588	1.000	0.000	Fixed	0.345
	sd3	0.582	0.990	0.102	<.001	0.338
	sd5	0.540	0.918	0.110	<.001	0.291
	sd6	0.615	1.047	0.085	<.001	0.378
	sd7	0.642	1.093	0.100	<.001	0.412
	sd8	0.750	1.277	0.114	<.001	0.563

	sd9	0.832	1.415	0.112	<.001	0.692
	sd11	0.893	1.519	0.119	<.001	0.797
IR	ir1	0.634	1.000	0.000	Fixed	0.402
	ir3	0.621	0.979	0.079	<.001	0.385
	ir4	0.763	1.204	0.089	<.001	0.582
	ir5	0.669	1.055	0.080	<.001	0.447
	ir8*	0.450	0.710	0.080	<.001	0.203
	ir9	0.856	1.351	0.095	<.001	0.733
	ir10	0.777	1.225	0.096	<.001	0.603
	ir11	0.720	1.136	0.095	<.001	0.519
SI	si1	0.740	1.000	0.000	Fixed	0.548
	si2	0.627	0.846	0.067	<.001	0.393
	si3	0.647	0.873	0.071	<.001	0.418
	si4	0.714	0.965	0.061	<.001	0.510
	si5*	0.507	0.684	0.074	<.001	0.257
	si6*	0.487	0.658	0.083	<.001	0.237
	si7	0.701	0.947	0.067	<.001	0.491
	si8	0.921	1.243	0.075	<.001	0.848
RI	ri2	0.677	1.000	0.000	Fixed	0.458
	ri3	0.733	1.083	0.086	<.001	0.537
	ri7	0.738	1.091	0.085	<.001	0.545
	ri8	0.819	1.210	0.094	<.001	0.671
	ri9	0.701	1.036	0.113	<.001	0.491
	ri10	0.864	1.277	0.092	<.001	0.746
	ri11	0.860	1.272	0.104	<.001	0.740
	ri12	0.759	1.122	0.095	<.001	0.576

Note. QoL = Quality of Life; β = standardized factor loading; *SE* = Standard error; r^2 = r squared; *p* = p value; (*) Denotes reverse item; MW = Material wellbeing; PW = Physical wellbeing; EW = Emotional wellbeing; PD = Personal development; SD = Self-determination; IR = Interpersonal relationships; SI = Social inclusion; RI = Rights.
