# Supplementary material for: Measuring the quality of life of students with autism in Chilean general education schools
Source: Front Psychiatry. 2026 May 20;17:1790139. doi: 10.3389/fpsyt.2026.1790139 (PMC13230136; doi:10.3389/fpsyt.2026.1790139)
Supplement: Supplementary file 4 [file Table4.pdf]

Table S4.

First order 8-8 model. Relationship between factors (QoL domains) according to the CFA (standardized covariances)

Factor	Factor	Standardized covariances
**MW**	PW	.84
	EW	.74
	PD	.73
	SD	.68
	IR	.68
	SI	.65
	RI	.72
**PW**	EW	.86
	PD	.82
	SD	.74
	IR	.73
	SI	.74
	RI	.82
**EW**	PD	.91
	SD	.79
	IR	.79
	SI	.69
	RI	.82
**PD**	SD	.72
	IR	.78
	SI	.69
	RI	.80
**SD**	IR	.84
	SI	.72
	RI	.72
**IR**	SI	.90
	RI	.79
**SI**	RI	.70

Note. MW = Material wellbeing; PW = Physical wellbeing; EW = Emotional wellbeing; PD = Personal development; SD = Self-determination; IR = Interpersonal relationships; SI = Social inclusion; RI = Rights.
